# Endothelial damage, vascular bagging and remodeling of the microvascular bed in human microangiopathy with deep white matter lesions

**DOI:** 10.1186/s40478-018-0632-z

**Published:** 2018-11-23

**Authors:** Karin M. E. Forsberg, Yingshuang Zhang, Johanna Reiners, Martina Ander, Alexandra Niedermayer, Lubin Fang, Hermann Neugebauer, Jan Kassubek, Istvan Katona, Joachim Weis, Albert C. Ludolph, Kelly Del Tredici, Heiko Braak, Deniz Yilmazer-Hanke

**Affiliations:** 1 Department of Medical Biosciences & Pathology, Umeå Universitet, Umeå, Sweden; 2 Department of Neurology, Peking University Third Hospital, Beijing, China; 3 Clinical Neuroanatomy Section, Neurology, School of Medicine, Ulm University, Helmholtzstr. 8/1, 89081 Ulm, Germany; 4 Department of Neurology, Ulm University Hospital, Ulm University, Ulm, Germany; 5 Institute of Neuropathology, School of Medicine, RTWH Aachen University Hospital, Aachen, Aachen, Germany

**Keywords:** Leukoaraiosis, Binswanger’s disease, Endothelial glycocalyx, Basement membrane, Blood brain barrier, Microglia activation, String vessel

## Abstract

White matter lesions (WMLs) are a common manifestation of small vessel disease (SVD) in the elderly population. They are associated with an enhanced risk of developing gait abnormalities, poor executive function, dementia, and stroke with high mortality. Hypoperfusion and the resulting endothelial damage are thought to contribute to the development of WMLs. The focus of the present study was the analysis of the microvascular bed in SVD patients with deep WMLs (DWMLs) by using double- and triple-label immunohistochemistry and immunofluorescence. Simultaneous visualization of collagen IV (COLL4)-positive membranes and the endothelial glycocalyx in thick sections allowed us to identify endothelial recession in different types of string vessels, and two new forms of small vessel/capillary pathology, which we called vascular bagging and ghost string vessels. Vascular bags were pouches and tubes that were attached to vessel walls and were formed by multiple layers of COLL4-positive membranes. Vascular bagging was most severe in the DWMLs of cases with pure SVD (no additional vascular brain injury, VBI). Quantification of vascular bagging, string vessels, and the density/size of CD68-positive cells further showed widespread pathological changes in the frontoparietal and/or temporal white matter in SVD, including pure SVD and SVD with VBI, as well as a significant effect of the covariate age. Plasma protein leakage into vascular bags and the white matter parenchyma pointed to endothelial damage and basement membrane permeability. Hypertrophic IBA1-positive microglial cells and CD68-positive macrophages were found in white matter areas covered with networks of ghost vessels in SVD, suggesting phagocytosis of remnants of string vessels. However, the overall vessel density was not altered in our SVD cohort, which might result from continuous replacement of vessels. Our findings support the view that SVD is a progressive and generalized disease process, in which endothelial damage and vascular bagging drive remodeling of the microvasculature.

**Electronic supplementary material:**

The online version of this article (10.1186/s40478-018-0632-z) contains supplementary material, which is available to authorized users.

## Introduction

Cerebral small vessel disease (SVD) or cerebral microangiopathy are overarching terms for a group of heterogeneous disorders with different etiologies and pathogeneses, which compromise the cerebral microcirculation [75]. SVD encompasses atherosclerosis and lipohyalinosis of small arteries and arterioles, cerebral amyloid angiopathy (CAA), hereditary forms of non-CAA microangiopathies (e.g., CADASIL), inflammatory angiitis, venous collagenosis, and miscellaneous forms, e.g., SVD that results from radiation or non-CAA-related vessel degeneration in Alzheimer’s disease. Pathologic alterations caused by sporadic and hereditary forms of SVD include white matter lesions (WMLs), lacunar infarcts, microinfarcts, and microbleeds [64].

WMLs, also referred to as leukoaraiosis, are large areas of pallor in the subcortical deep white matter and in periventricular areas with ill-defined margins that show hyperintense signals in T2-weighted magnetic resonance images [61]. They are frequently found in the healthy, elderly population, and their prevalence increases from 11 to 21% in adults around the age of 64 to 94% in those around 82 years of age [34, 62, 87]. WMLs are largely considered a clinically silent brain injury, but their presence increases the risk of developing stroke and vascular mortality [28, 46, 47, 82]. WMLs are also commonly associated with cognitive decline and dementia [33, 74], general brain atrophy [5], and gait disorders [26, 81], often leading to the diagnosis of Binswanger’s disease, particularly in the presence of lacunar infarctions [43]. The pathogenesis of WMLs has not been fully established yet, but the prevailing view is that they result from white matter ischemia owing to hypertension or chronic hypoperfusion [10, 64, 70].

Pathological changes found in WMLs such as hyalinosis of small arterioles, arteriolosclerosis, and arterial tortuosity are widespread in the elderly population and in hypertension, probably contributing to disturbances in the perfusion of deep white matter areas [17, 32, 58, 65]. Nevertheless, in some stroke patients with WMLs no subcortical arteriosclerotic changes have been detected [48]. In the human brain, deep white matter areas that are particularly vulnerable to injury from hypoperfusion are the so-called watershed areas, which are located at the border between territories supplied by terminal branches of leptomeningeal and perforating arteries [57, 64]. In experimental models, the predominant microvascular pathology in chronically hypoperfused white matter areas is endothelial cell damage [42, 78]. Reduced expression of endothelial markers and leakage of plasma proteins into arteriole walls and the white matter have also been reported in human WMLs [15, 84, 89]. Further histopathological features of human WMLs include dilated perivascular Virchow-Robin spaces, loss of oligodendrocytes leading to demyelination, axonal damage and vacuolization (spongiosis) of the white matter, and potentially alterations in the density of so-called string vessels, which are collagenous tubes connecting two vessels and are regarded as remnants of basement membranes in regressing vessels [18, 19, 21, 61].

The aim of this study was to investigate markers and mechanisms that may be involved in remodeling of vessels in SVD patients with WMLs. Vascular bagging, defined here as the space between the vessel wall and external collagenous membranes of small vessels, as well as string vessels were analyzed at lesion sites and in control regions using double-labeling for endothelial and basement membrane markers. Using Z-stack imaging, different types of string vessels as well as the relationship of activated IBA1- and CD68-positive cells to the cerebral microvasculature were studied in thick sections. Widened perivascular spaces, also called état criblé or status cribrosus, and plasma protein leakage to the vessel walls or brain parenchyma were examined in thin paraffin sections. Hereby, we focused on deep WMLs (DWMLs), because periventricular WMLs that are often associated with prominent fibrosis in the wall of periventricular veins, also called venous collagenosis [58], might be assignable to a different pathological entity [35]. Particular attention was also paid to excluding concomitant pathologies related to Alzheimer’s and Parkinson’s disease or other tauopathies and alpha-synucleinopathies, which can affect the microvascular bed [19, 86]. Moreover, SVD cases with vascular brain injury (VBI) were studied separately from “pure” SVD cases without VBI, because especially in the acute/subacute phase cerebral infarctions can lead to the infiltration of the brain with peripheral blood cells [9], potentially confounding findings related to chronic alterations in SVD.

## Material and methods

### Study population and neuropathological evaluation of the brain

The study cohort consisted of brain tissue from 14 human subjects (7 females, 7 males) with an average age of 64.1 ± 10.2 years (mean ± standard deviation, SD). Demographics and relevant data for the patient cohort are provided in (Additional file 1: Table S1). The patients were divided into 3 groups: “pure” SVD cases with DWMLs but without additional VBI (*n* = 5), SVD + VBI cases with DWMLs and remote cerebrovascular incidents (*n* = 5), and control cases without SVD (NoSVD) or history of neurological disorders (*n* = 4). NoSVD controls died from myocardial infarction, ovarian cancer, esophageal cancer, and pulmonary edema due to left heart failure. Among cases with pure SVD, one case suffering from chronic hypertension and generalized arteriosclerosis died following aortic valve replacement surgery (24 h blood pressure levels of 149/70 mmHg under a combination therapy with the diuretic hydrochlorothiazide, the beta-blocker metoprolol, the calcium channel blocker amlodipine, and the angiotensin receptor 1 blocker valsartan). A second case had advanced peripheral artery disease at Fontaine stage IIb to III and died from a gastrointestinal infarct due to occlusion of the pelvic artery. The other three pure SVD cases were diagnosed with pulmonary embolism, highly malignant non-hodgkin lymphoma and renal cancer, respectively. In three SVD + VBI cases, pontine bleeding and a contralateral (sub)acute ischemic stroke (MCA-l) with/without thalamic infarction was the cause of death. An SVD + VBI case with ischemic stroke in the contralateral internal capsule died from breast cancer and the other case with a chronic ischemic cerebral infarct (2 years old) from sudden cardiovascular arrest. All autopsied subjects underwent routine neuropathological examination and were screened for tauopathies, alpha-synucleinopathies and beta-amyloid (Aβ) deposition. Inclusion criteria for entering the study were: No history of neurodegenerative disorders; i.e., no Alzheimer-related tau pathology exceeding neurofibrillary stage II [13] or other tauopathy, as well as no Parkinson’s disease [14], multisystem atrophy or other alpha-synuclein-related pathology. Furthermore, all cases included to the study had Aβ phases II or less [76]. This retrospective study was performed in compliance with the university ethics committee guidelines as well as German federal and state law governing human tissue usage. Informed written permission was obtained from all patients and/or their next of kin for autopsy.

### Histology

Brains fixed in a 4% solution of formaldehyde were cut in approximately 1 cm thick coronal slices. Tissue blocks containing frontoparietal and temporal lobe areas that were devoid of macroscopically visible small or large infarcts or cysts in the white matter were embedded in polyethylene glycol (PEG 1000, Merck, Carl Roth Ltd., Karlsruhe, Germany). Multiple 100 μm thick consecutive sections were obtained using a sliding microtome (Jung, Heidelberg, Germany). For histological orientation, sections were stained for lipofuscin pigment and the Nissl substance using aldehyde fuchsine and Darrow red [12]. For neuropathological evaluation and the identification of DWMLs, thick coronal sections were stained with a modified hematoxylin eosin (H&E) procedure by replacing eosin with acid fuchsine, which allowed examination of entire hemisphere sections [60]. Paraffin embedding was performed on tissue blocks dissected out of deep white matter areas neighboring the regions that have been analyzed in thick coronal sections. From paraffin embedded blocks, 7 μm thick sections were cut with a microtome (Slee Medical GmbH, Mainz, Germany). Myelin staining was performed with a modified Heidenhain procedure using 2.5% ammonium iron(III) sulfate (30 min) and a solution containing 9% hematoxylin and 0.03% lithium carbonate (60 min) [44].

Upon neuropathological evaluation, arteriolosclerotic changes were found in the basal ganglia of the case with hypertension (pure SVD), which showed prominent concentric hyaline thickening of vessel walls and stenosis of the vessel lumen, and two other cases also showed minor hyalinosis (NoSVD control; SVD + VBI). However, vessels with an “onion-skin” pattern and Charcot-Bouchard microaneurysms were not observed. SVD cases with VBI also presented subcortical microbleeds (4 out of 5 cases). Particularly enlarged perivascular spaces were found in the basal ganglia and thalamus of 4 SVD cases around vessels of various calibers (2 pure SVD; 2 SVD + VBI), although the NoSVD controls and other SVD cases also showed some widening of perivascular spaces.

### Immunohistochemistry in thick sections

Free-floating 100 μm thick sections were treated for 30 min with a mixture of 10% methanol and 3% concentrated H_2_O_2_ in Tris-buffered saline (TBS) to inhibit endogenous peroxidase activity. Bovine serum albumin (BSA) was applied for 30 min for blocking non-specific binding sites. Antigen retrieval was performed using Tris-EDTA buffer at pH 9.0 or citrate buffer at pH 6.0 for 1/2 h at 100 °C or pretreatment with 1.3 μg/ml proteinase K for 10–15 min at 37 °C (Invitrogen/Life Technologies, Darmstadt, Germany). For single labeling, sections were incubated at 4 °C with the primary antibody for a duration of 12–48 h (depending on the antibody). The three primary antibodies used were directed against collagen IV (COLL4; 1:5000, rabbit, Abcam, Cambridge, UK), the microglia/macrophage marker ionized calcium binding adapter molecule 1 (IBA1; 1:5000, rabbit, Abcam, Cambridge, UK) or the macrophage marker from the lysosomal/endosomal-associated membrane glycoprotein (LAMP) family CD68 (1:200, mouse, DAKO, Glostrup, Denmark). Sections were transferred for 2 h to a solution containing the corresponding secondary biotinylated antibody (1:200; Vector Laboratories, Burlingame, CA, USA). Alternatively, sections were incubated for 48 h with *Ulex europaeus* lectin (UEA-l; 1: 800, biotin-coupled, GeneTex, Irvine, CA, USA). Immunohistochemical reactions or lectin binding were visualized by incubating the sections for 2 h with an avidin-biotin-peroxidase complex (ABC Vectastain, Vector Laboratories, Burlingame, CA, USA). The reaction product of the peroxidase was visualized with the chromogen 3,3′-diaminobenzidine tetrahydrochloride (DAB; Sigma Taufkirchen, Germany). For double-label immunohistochemistry, sections were washed with TBS at 95 °C for 5 min, and the immunohistochemical procedure was repeated using the next primary and secondary antibodies. Subsequently, a blue chromogen (Vector SK-4700 peroxidase substrate kit, Linaris, Doffenheim; Germany) was used to visualize the reaction product. Omission of the primary antibody resulted in non-staining.

### Immunohistochemistry and immunofluorescence in paraffin sections

Thin paraffin sections were treated with 10% methanol and 3% H_2_O_2_ in TBS for 30 min and/or with BSA for 30–60 min. For antigen retrieval, Tris-EDTA or citrate buffer were used at 100 °C for 10–20 min or proteinase K was applied for 10–15 min as described above. For immunohistochemistry (IHC) and immunofluorescence (IF), sections were incubated with primary antibodies against COLL4 (IHC: 1:5000, IF 1:4000, rabbit, Abcam), alkaline phosphatase (ALPL; IHC 1:1000, rabbit, Atlas Antibodies, Sweden), fibrinogen (FIBR; IHC 1:200, rabbit, DAKO), human IgG (IHC/IF 1:200, Vector Laboratories) or myelin basic protein (MBP; IHC 1:1000, rat, BioRad, Puchheim, Germany). For IHC, sections were treated with a biotinylated secondary antibody (1:200 for 2 h, Vector Laboratories, Burlingame, CA, USA) or UEA-l (1:800 for 48 h, GeneTex) and transferred to a ABC Vectastain solution for 2 h. The reaction product was visualized with DAB, SK-4700 or SK-4800 (Vector Laboratories), and the sections were coverslipped. For IF, binding of UEA-l (1:100, biotin-coupled, overnight) or primary antibody was visualized by incubating sections with streptavidin coupled to Alexa 532 (1:1000, Invitrogen/Life Technologies) or with a secondary antibody (Abcam) coupled to Alexa 594 (1:200, anti-rabbit) or Alexa 647 (1:300, anti-goat). Sections were coverslipped with Moviol (Polysciences Europe, Hirschberg an der Bergstrasse, Germany). Omission of primary antibodies resulted in absence of IHC and IF.

### Image acquisition and processing

Alterations in the microvascular bed and microglia activation were assessed qualitatively and quantitatively with the aid of a AX10 microscope (Zeiss, Jena, Germany). Digital micrographs were taken with a Jenoptik Progres Gryphax® Prokyon camera using the Progres Gryphax® microscope camera software (Jena, Thüringen, Germany). In IHC-stained sections, either single images were taken or z-stacks were obtained. For documentation of tortuous vessels and pathological alterations, multiple single images and z-stacks were combined using manual construction with Adobe Photoshop, version 10.0 for qualitative analyses as needed. Microscope and camera settings (exposure, gain, and hue) were held constant when taking images for quantitative analyses. For IF applications, sections were imaged with a LED fluorescence lamp and narrow band filter sets (AHF Analysetechnik, Tübingen, Germany). Consistency of immunohistochemical staining throughout 100 μm thick sections was verified by confirming staining at different focus levels (see Additional files 2 and 3: Videos S1 and S2 showing videos of vessels and microglia). For video-documentation, an Eclipse LV100ND microscope was used that was equipped with a digital DS-Fi3 camera and the NIS-Elements software (NIKON GmbH, Düsseldorf, Germany) and with a motorized object table (Märzhäuser Wetzlar, Wetzlar, Germany).

### Quantification of vessel densities, vessel diameters and microglia

Quantitative analyses were performed in 100 μm thick sections using the Image J software version v1.51 k (NIH, Bethesda, Maryland, MD, USA). The density and diameter of vessels were quantified in thick sections double-labeled for UEA-l and COLL4, and the density of central nervous system (CNS) macrophages in sections double-labeled for CD68 and COLL4 by a blind investigator. The boundaries of DWMLs and in-case control areas (approx. 1 cm^2^) were marked on the immunostained sections after identifying pale white matter areas in adjacent sections stained for modified H&E. Depending on the localization of the lesion site, in-case control areas were positioned in the medial or lateral frontoparietal region, and an additional remote control area in the lateral temporal lobe was included. All areas studied were located in the subcortical deep white matter proximal to U-fibers.

Vessel densities were measured within the marked white matter area in images taken with the 5x objective. After transforming each image into an 8-bit gray image, the distribution of gray values and the standard deviation (SD) were determined. From these 8-bit gray images, binary images were obtained using an established pipeline by first subtracting the background (mean gray value minus 2x SD) and then by median filtering (1.5 px range). After superimposing a grid on the binary image, the vessel density was measured in every second grid box (area 0.3025 mm^2^) by selecting the grid boxes in a checkerboard pattern. Grid boxes containing arteries or veins were skipped by moving to the next available grid box. Altogether, 35 grids were analyzed in NoSVD controls, 74 grids in pure SVD, and 70 grids in SVD + VBI. In addition, string vessels with different morphologies were counted by screening the white matter with the 10x objective. The number of string vessels counted was divided by the size of the area screened to calculate the density of string vessels. Overall, the density of string vessels was determined in an area of 8.905 mm^2^ in NoSVD control cases, of 9.577 mm^2^ in pure SVD cases and of 7.603 mm^2^ in SVD + VBI cases. For quantification of vessel diameters, images of vessels were taken with the 20x objective at a distance of 1 mm in both the x- and y-axes. In these images, vessel diameters were measured by selecting vessel segments that were in focus (all vessel components sharp and clearly discernable). The regular diameter of the vessels (UEA-l- and COLL4-labeled) and the maximum outer diameter (at the COLL4-labeled outer vascular bag membrane) were measured. In addition, the length of each vessel segment was determined, in which the two vessel diameters were recorded. The calculated difference between the maximum outer diameter and corresponding actual vessel diameter was used as an indicator of vascular bagging. Overall, vessel diameters were analyzed in 2709 vessel segments with an average length of 93.45 ± 75.8 μm (SD) per vessel segment, thereby resulting in a total vessel length of 253,152 μm.

CD68-positive cells (macrophages) were quantified in sections double-labeled for COLL4. Lesion and control white matter areas were marked on the sections by using neighboring sections stained for modified H&E to identify the DWML. Z-stack images were taken with the 20x objective throughout the entire section. The images covered an area of 0.495 μm^2^ in the xy-plane and had a distance of 1 mm in the x- and y-axes. The area of the cell body was measured manually for each CD68-positive cell in the z-stack images. Crests/cups of cell bodies cut at the surface or bottom of the section (< 20 μm^2^ area) were excluded from analyses. The number of cells and the area of cell bodies were quantified in the parenchyma as well as at perivascular sites by identifying cells attached to the outer wall of COLL4-positive vessels. Cell densities (cell counts / image area) were calculated for each image taken. The number of images analyzed was 128 in the NoSVD controls, 153 in the pure SVD group, and 122 in the SVD + VBI group.

### Statistical analysis

Statistical analyses were performed with the software IBM SPSS Statistics Version 25. Small vessels included in the statistical analyses were identified by determining large vessels with extreme values using explorative descriptive statistics, thereby permitting the exclusion of outliers with a vessel diameter above the 95th percentile. For comparing two groups, a t-test was performed (Welch test with Satterthwaite’s approximation to compute the degrees of freedom). Multiple groups were compared using generalized linear models for the main factors vascular disease (NoSVD control, pure SVD or SVD + VBI), presence of DWMLs (NoSVD control, in-case control or DWMLs) and white matter location (frontoparietal or temporal) and covariate age with the aid of a three-way ANOVA or one-way ANOVA followed by the posthoc Games-Howell test. Data were presented as mean ± S.E.M. and outcomes were deemed significant at a two-tailed level of *p* < 0.05.

## Results

### DWMLs showed pallor, spongiosis and reduced oligodendrocyte densities

**Fig. 1:**
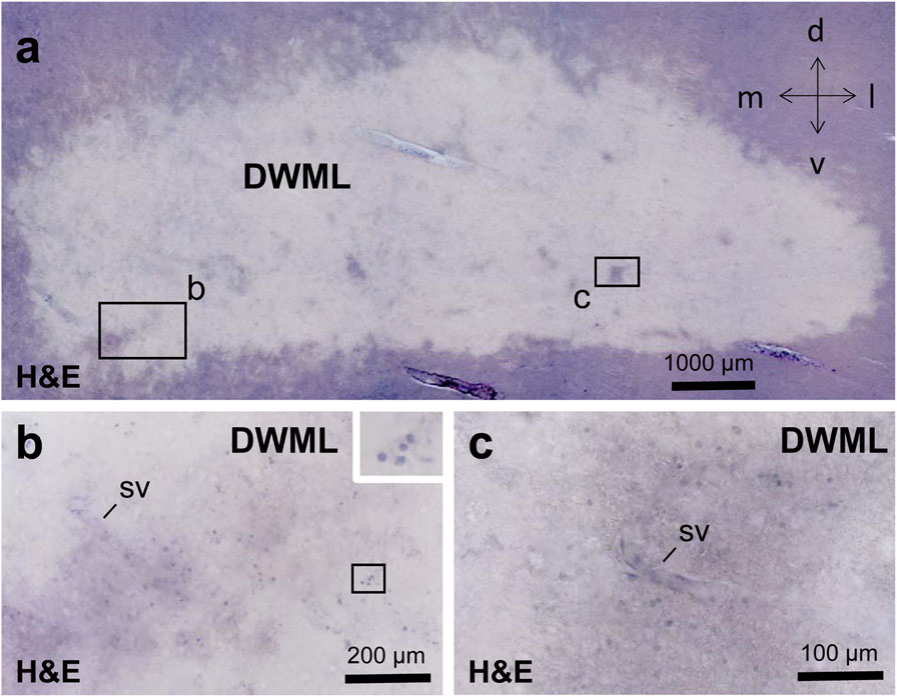
Example of deep white matter lesion (DWML) in a case with small vessel disease (SVD) in thick sections (**a**-**c**). **a** Pale DWML with unclear boundaries and islands with less pale areas. **b**-**c** Insets from (**a**) show more oligodendrocytes in relatively well-preserved less pale areas compared to severely affected pale areas, and also small vessels (sv). Case 6 (non-hodgkin lymphoma) with “pure” SVD, i.e., this case has only SVD, but no additional vascular brain injury (VBI) resulting from a ischemic or hemorrhagic cerebral infarct. Scale bars: 1000 μm in (**a**), 200 μm in (**b**), 100 μm in (**c**)

The DWMLs analyzed in the present study were situated in the frontoparietal region, with some lesions extending into the dorsal portion of the internal capsule except for a single DWML area that was located in the temporal lobe. In thick coronal sections stained with modified H&E, DWMLs could be identified as pale areas separated from the apparently normal surrounding tissue (Fig. 1a). At higher magnification, pale areas within DWMLs showed a patchy staining pattern, and contained a variable density of putative oligodendrocytes with small, round and heterochromatin-rich nuclei. However, cavitation and tissue loss were not evident in the white matter areas studied (Fig. 1b-c). In thin paraffin sections, IHC for MBP and myelin staining revealed loosening of the white matter architecture with spongiosis and a reduced density of oligodendrocytes in DWML areas. In contrast, NoSVD controls exhibited dense bundles of fiber tracts with clear boundaries that ran in various directions and had a high density of oligodendrocytes (Additional file 4: Figure S1).

### Vascular bagging was revealed as a hallmark of white matter pathology in SVD

**Fig. 2:**
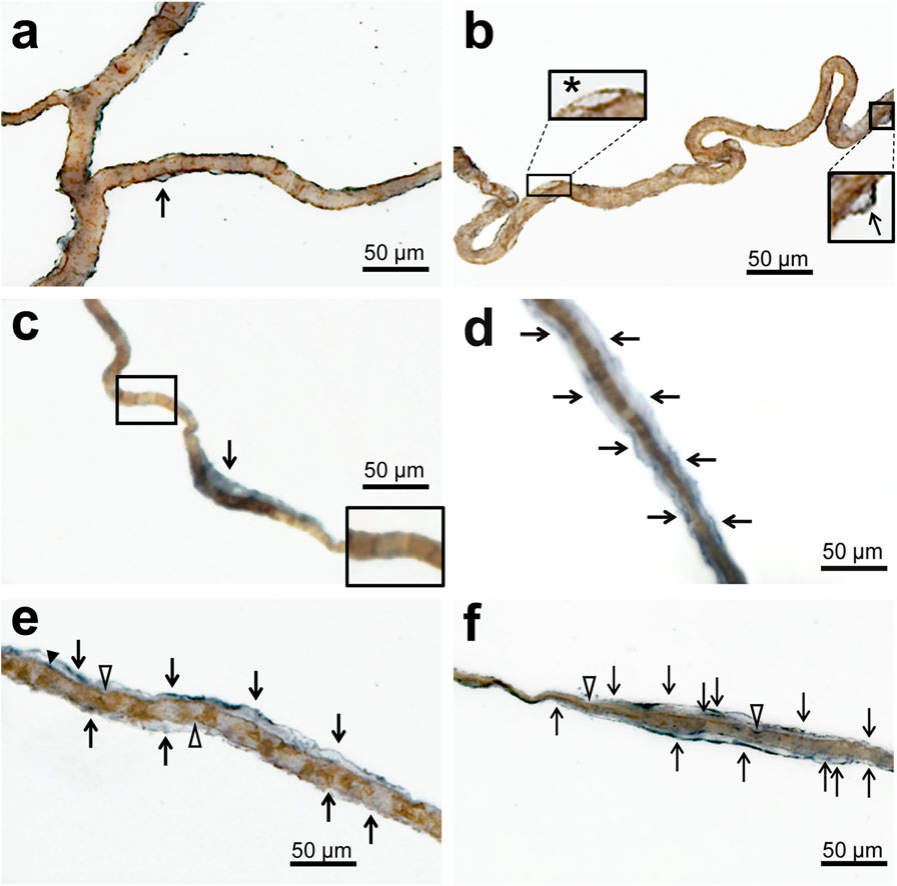
Z-stack images of vessels double-labeled for the endothelial marker *Ulex europaeus* lectin (UEA-l, brown) and collagen IV (COLL4, blue) in 100 μm thick sections. **a**-**b** In healthy vessels (type 1), COLL4-positive membranes are only visible in areas with bubble-shaped expansions (arrows) as seen in a control case without SVD (NoSVD). Small endothelial indentations are also found in healthy vessels (star; type 2a), which have COLL4-positive basement membranes with smooth contours (see Fig. 6b for comparison with type 2b vessel). **c**-**d** COLL4- positive basement membranes of small vessels are often barely distinguishable from the staining of the endothelium (labeled with UEA-l) in healthy vessels (inset in **c**) in thick sections except in vessel segments with vascular bags. Vascular bags (arrows) are formed by external COLL4-positive membranes detached from the vessel wall. In NoSVD controls they are usually limited to short vessel segments (type 3a, see **c**), whereas in SVD they can cover long distances (type 3b, see **d**). **e**-**f** Severely diseased vessels (type 4) show both endothelial irregularities (open arrow heads) and vascular bagging with thickened basement membranes (small black arrow head). Vascular bagging can extend over considerable distances in type 4 vessels with various diameters (arrows) in SVD. Double arrows indicate multiple layers of collagenous membranes. Images obtained from Case 1 (myocardial infarction, NoSVD) (**a**-**b**), Case 2 (ovarian cancer, NoSVD) (**c**-**d**), and Case 6 (non-hodgkin lymphoma, pure SVD) (**e**-**f**). For viewing vascular bags, also see Additional file 2: Video S1 of Vessels. Scale bars 50 μm

Double-labeling for UEA-l and COLL4 studied allowed the visualization of the endothelial cell layer and basement membrane of vessels, respectively. Analyses of large white matter areas in thick sections revealed pouches formed by COLL4-positive membranes around many vessels, which we designated as ‘vascular bagging’ (Fig. 2). The endothelium occasionally showed indentations in the absence of abnormalities in the basement membrane (Fig. 2b). However, vascular bags with irregular expansions of COLL4-positive outer membranes were more common. They often formed multiple layers attached to the vessel surface, suggesting duplication or multiplication of the basement membrane. Such external vascular bags largely differed in their size and extent, and some vascular bags accompanied the affected vessels for distances in excess of several hundred micrometers in DWMLs (Fig. 2c-d and Fig. 6b).

Based on these observations, four types of morphologies were identified in small vessels or vessel segments (Additional file 5: Figure S2). Type 1 normal vessels had an intact endothelium labeled with UEA-l and a smooth COLL4-positive basement membrane lying directly underneath the endothelium and tightly attached to it. These vessels were the most common type in our study population. Type 2 vessels, which showed an intact basement membrane but indentations or irregularities of various degrees in their endothelial layer, were comparatively rare. Type 3 vessels had COLL4-positive bags attached to the vessel wall that were formed by external collagenous membranes and resembled small pouches or long tubes. However, the vessel wall was smooth and the endothelium with its underlying basement membrane appeared intact. Type 4 vessels displayed irregularities in both the endothelial layer and external collagenous membranes (bagging), which sometimes consisted of multiple layers or were discontinuous.

### Vascular bagging was more prominent in DWMLs and age-dependent in control areas

**Fig. 3:**
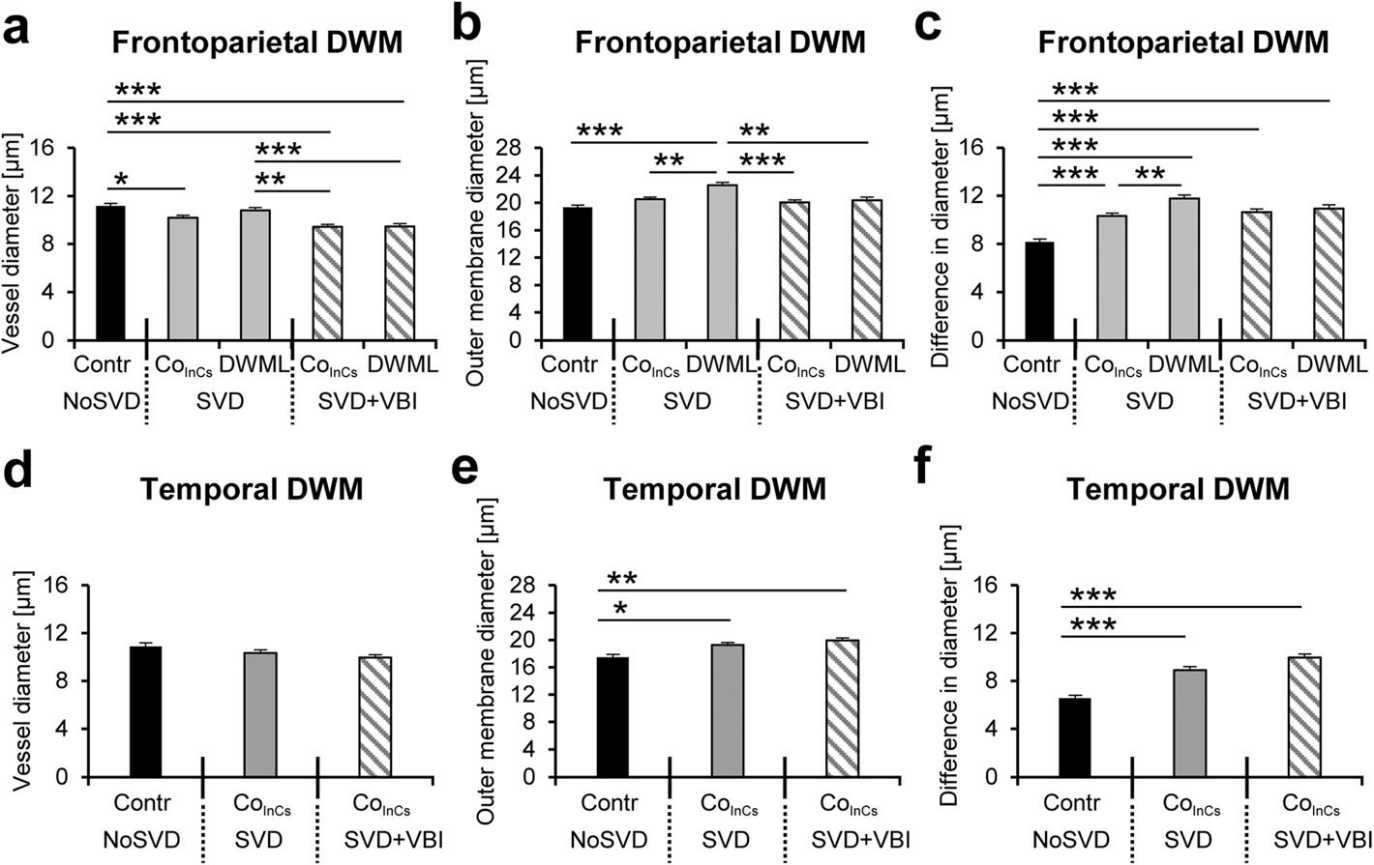
Quantification of vascular bagging (**c** and **f**), which was defined as the difference between the diameter of the vessel lumen (**a** and **d**) and outer COLL4-positive bag membrane (**b** and **e**). In general, vessel diameters are larger and vascular bagging is more severe in the deep white matter (DWM) of frontoparietal areas than in the temporal lobe. **a** and **d** Vessel calibers are slightly smaller in the in-case control area of “pure” SVD cases and all white matter areas of SVD + VBI cases in the frontoparietal region compared to NoSVD controls (**a**), but no differences are seen in the temporal lobe (**d**). **b** and **e** In the frontoparietal region, the outer membrane diameters (measured at the outer border of bags) are significantly higher in DWMLs of “pure” SVD cases than in all other groups studied. In the temporal lobe, the in-case control areas exhibit significantly increased outer membrane diameters in both “pure” SVD and SVD + VBI cases when compared to NoSVD controls (**b**). **c** and **f** In the frontoparietal region, vascular bagging is more severe in SVD (both pure SVD and SVD + VBI) in all regions studied (DWML and in-case control) than in NoSVD cases. Furthermore, in "pure" SVD cases vascular bagging is significantly increased in DWMLs when compared to in-case control areas (**c**). In the temporal lobe, in-case control areas of SVD cases show a significant increase in vascular bagging when compared to NoSVD control cases, indicating a generalized disease process (**f**). The “pure” SVD cases were referred to as SVD in the diagrams. * *p* < 0.05, ** *p* < 0.01, *** *p* < 0.001

Vascular bags consisting of the space between the basement membrane of the UEA-l labeled endothelium and the external COLL4-positive membranes forming the outermost vessel boundaries were quantified in small vessels using thick sections. Large vessels with diameters above 95th percentile (> 22.7 μm) were excluded from analyses (Additional file 6: Figure S3). The effect of vascular disease, presence of DWML, and white matter location on vessel diameters, the outer membrane diameters and vascular bagging (difference between the two diameters) were analyzed with the aid of three-way ANOVA (Fig. 3). Results indicated that vascular disease had a significant effect on the vessel diameters (F_2,2700_ = 17.927, *p* < 0.001) and outer membrane diameters of small vessels (F_2,2700_ = 16.056, *p* < 0.001). In addition, the presence of a DWML had a significant effect on vascular bagging (F_2,2700_ = 5.836, *p* < 0.05). Posthoc analyses revealed that the vessel diameters in the frontoparietal white matter were significantly reduced in SVD + VBI cases when compared to NoSVD controls (Fig. 3a). In contrast, the outer membrane diameters were significantly increased in the DWMLs in pure SVD compared to white matter areas of all other groups (Fig. 3b). Vascular bagging was also increased significantly in the frontoparietal white matter of both SVD groups (pure SVD, SVD + VBI) compared to NoSVD controls, and this was not confined to DWMLs and included in-case control areas, indicating a generalized disease process in widespread white matter areas in SVD. Moreover, in pure SVD vascular bagging was more prominent in the DWMLs than in in-case control sites, compatible with a more advanced disease state in DWMLs in this vascular disease group (Fig. 3c).

Three-way ANOVA further showed that the white matter location (frontoparietal versus temporal) had a significant effect on the vessel diameters (F_2,2700_ = 4.247, *p* < 0.05), outer membrane diameters (F_2,2700_ = 11.085, *p* = 0.001), and vascular bagging (F_2,2700_ = 7.551, *p* < 0.01), combined with a significant interaction between presence of DWML and white matter location for the vessel diameters (F_2,2700_ = 7.069, *p* < 0.01). However, posthoc analyses only confirmed significantly smaller outer membrane diameters and less vascular bagging in the temporal lobe when compared to frontoparietal areas of NoSVD controls and SVD cases. Nonetheless, outer membrane diameters and vascular bagging were also increased significantly in the temporal lobe of SVD cases compared to NoSVD controls (Fig. 3e-f), whereas the actual vessel diameters were not affected (Fig. 3d). In the three-way ANOVA (vascular disease x presence of DWML x white matter location), the covariate age had a significant effect specifically on outer membrane diameters (F_1,2700_ = 101.374, *p* < 0.001) and vascular bagging (F_2,11_ = 166.641, *p* < 0.001), although the individuals from the different vascular disease groups did not significantly differ in their age as confirmed by one-way ANOVA (F_2,11_ = 0.33, *p* > 0.10).

### Vascular bags contained leaked plasma proteins

**Fig. 4:**
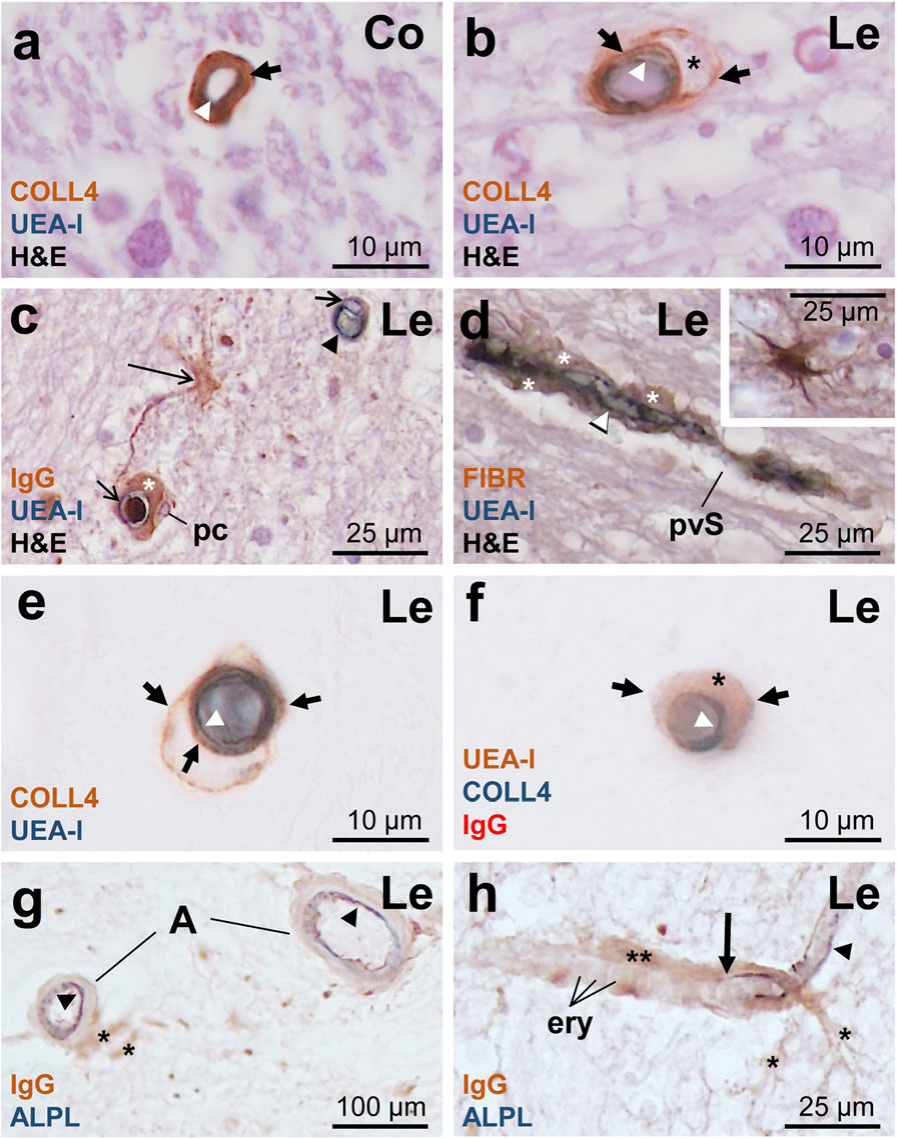
Double- and triple-label immunohistochemistry (IHC) for UEA-l, COLL4, the plasma proteins fibrinogen (FIBR) or immunoglobulin G fraction (IgG) and the endothelial marker alkaline phosphatase protein (ALPL) in 7 μm thick paraffin sections. **a**-**b** The healthy capillary in the control white matter (Co) of a NoSVD case does not have vascular bags (**a**). Vascular bag (short thick arrow) in a SVD case with multiple layers of COLL4-positive membranes around the UEA-l-labeled endothelium (white arrowhead) of a small vessel (< 10 μm diameter) at a lesion (Le) site. Hematoxylin eosin (H&E) counterstaining reveals eosinophilic material (star) in the vascular bag (**b**). **c**-**d** Capillaries with a UEA-l positive endothelium (white arrowhead) and endothelial cell nuclei (short thin arrows) are surrounded by pericytes (pc) and a sharply delineated area filled with plasma proteins IgG or FIBR (star) suggestive of vascular bagging. Plasma proteins also leak to the white matter parenchyma (large thin arrow), but the widened perivascular space (pvS) outside the vascular bag appears empty. **e**-**f** Vascular bags with COLL4-positive walls around the UEA-l positive endothelium are filled with IgG protein (star). **g**-**h** ALPL expression is found in the endothelium of small arteries (**a**) and arterioles (black arrowheads), but is lost at the transition from the pre-capillary arteriole to the capillary (large thick arrow). IgG accumulation found around the pre-capillary arteriole and capillary with erythrocytes (ery) extends into the nearby white matter (stars). Images obtained from Case 2 (ovarian cancer, NoSVD) (**a**), Case 13 (contralateral subacute ischemic stroke in the territory of the medial cerebral artery (MCA) with thalamic infarction, SVD + VBI) (**b**-**e**), and Case 6 (non-hodgkin lymphoma, pure SVD) (**f**-**h**). The list of (immuno)histochemical markers is provided on the lower left corner of each image (color-coded for the chromogen used). Uppermost marker: brown (Diaminobenzidine, DAB); Middle (or lower in double-label IHC) marker: blue (SK-4700, Vector Laboratories); Lower stain/marker: H&E or red (SK-4800, Vector Laboratories); ery erythrocyte

**Fig. 5:**
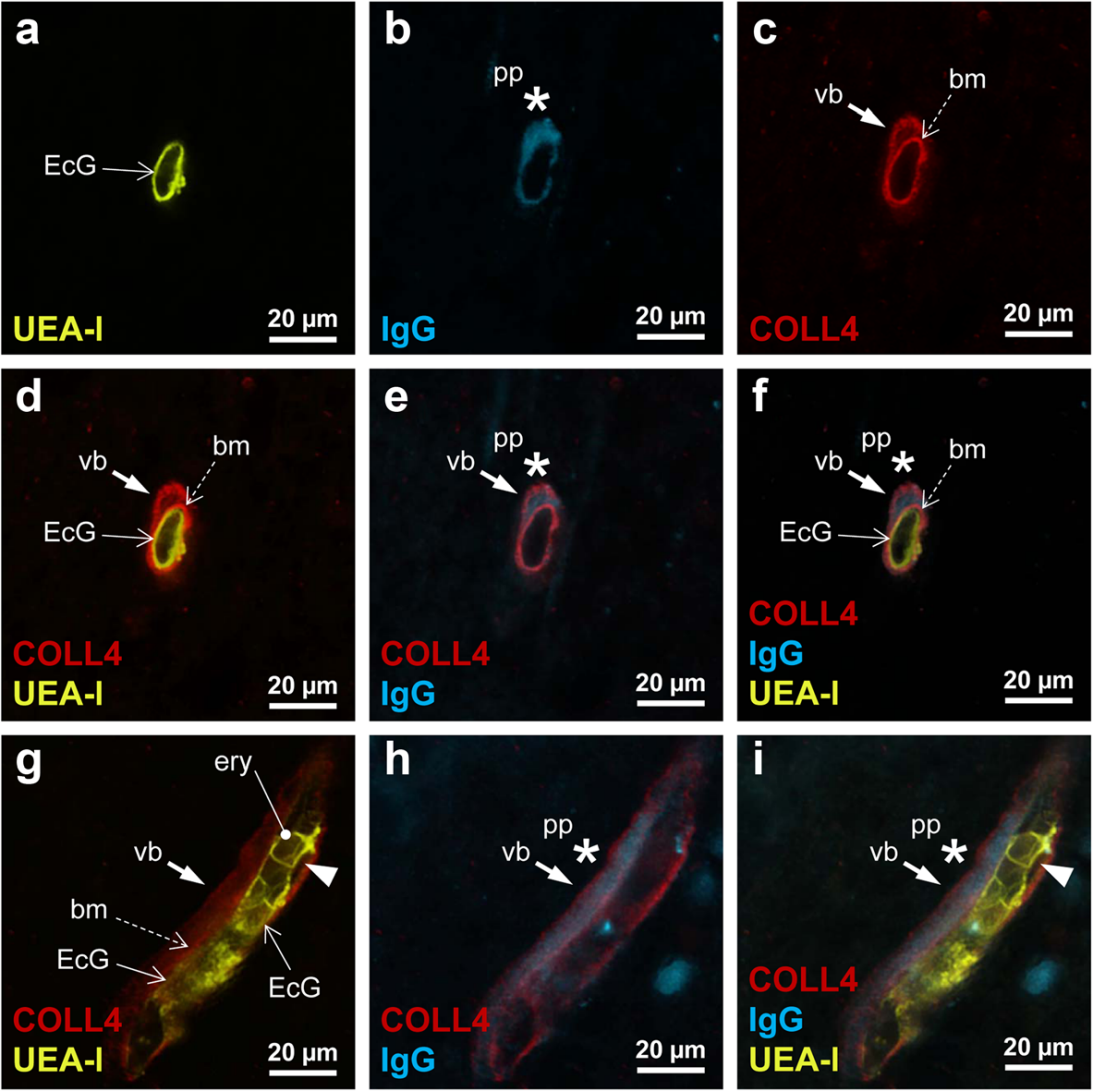
Triple-label immunofluorescence showing the location of the plasma protein (pp) IgG in relation to the endothelial cell glycocalyx (EcG) labeled with UEA-l, and COLL4-positive basement membrane (bm) and wall of vascular bag (vb). **a**-**f** Transverse or oblique section through small vessel with a smooth endothelial cell layer (continuous open arrow) and vascular bag (closed arrow), which is attached to the basement membrane (broken open arrow) and contains IgG (star). **g**-**i** Longitudinal section through a capillary-size small vessel shows the UEA-l-labeled EcG (open arrows) and erythrocytes (ery). The tubular vascular bag (closed arrow) around the vessel, which is filled with IgG (star), is formed by a COLL4-positive external membrane and the basement membrane (broken open arrow). An endothelial indentation with detachment from the basement membrane (arrow head) is also seen, but there is no IgG deposition between the endothelial layer and basement membrane. Case 12 (pontine bleeding, SVD + VBI)

Multiple-label IHC and IF were employed in thin paraffin sections to investigate vascular bagging in more detail (Figs 4 and 5). In the H&E stain, the eosinophilic collagenous membranes forming the wall of bags and their eosinophilic contents were not distinguishable from the adjacent white matter. However, IHC combined with H&E showed that COLL4-positive basement membranes tightly covered the UEA-l labeled endothelium of capillary-size small vessels in the white matter of NoSVD controls (Fig. 4a), whereas vessels in DWMLs had vascular bags with multiple layers of external COLL4-positive membranes filled with eosinophilic material (Fig. 4b). Further analyses showed that the vascular bags contained the plasma proteins FIBR and IgG, whereas pericyte nuclei and enlarged perivascular spaces were situated outside the bags (Fig. 4c-f). Double-label IHC for plasma proteins and ALPL, an endothelial marker highly expressed in arteries and arterioles [59], showed strong IgG and FIBR accumulation in the vascular bags around capillaries and ALPL-positive pre-capillary arterioles, from where the plasma proteins spilled to the white matter parenchyma. In contrast, IgG and FIBR levels were low in the media of larger arterioles (Fig. 4g-h). Triple-label IF confirmed the presence of a basement membrane at the inner (endothelial) side of vascular bags (Fig. 5). The basement membrane lying directly underneath the endothelium was clearly separable from the UEA-l labeled glycocalyx expressed on the endothelial surface (Fig. 5d). The outer collagenous membranes of vascular bags were directly attached to the basement membrane, suggesting a splitting or duplication of the membrane (Fig. 5c). While vascular bags with COLL4-positive walls were loaded with plasma proteins, no IgG or FIBR accumulation could be detected in the subendothelial space on the luminal side of the basement membrane (Fig. 5i).

### White matter regions showed four types of string vessels - S1-S4

**Fig. 6:**
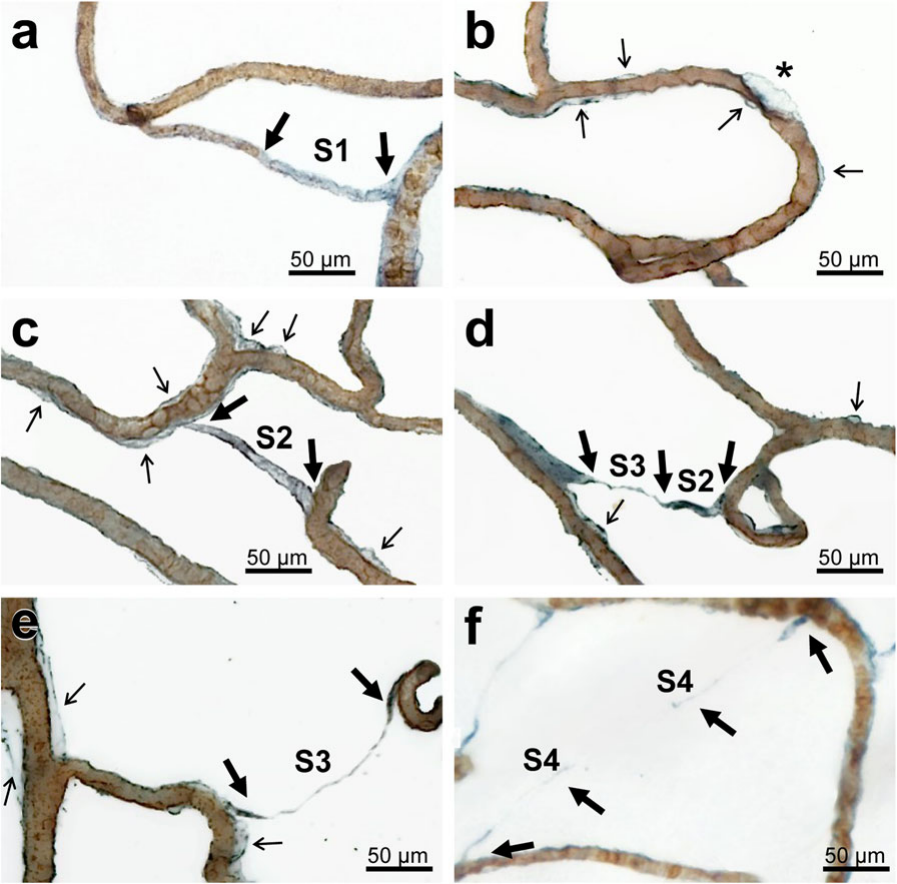
Z-stack images obtained from 100 μm thick sections double-labeled for the endothelial marker UEA-l (brown) and COLL4 (blue) showing endothelial damage and various stages of string vessels. **a** Gradual loss of the endothelium in an early string vessel (S1 type) that connects two other vessels. **b** Focal endothelial damage with a large COLL4-positive bag (star) in the vicinity of areas with vascular bagging (thin black arrows). **c** Early string vessel of the S2 type with an empty collagenous tube that connects two vessels but completely lacks endothelial lining. **d** String vessel with a tube section (S2 type) and with a thin collapsed collagenous section classified as S3 type. **e** Fully collapsed string vessel (S3 type) between two vessels. **f** Segmented string vessel with extremely thin or broken sections (S4 type), which was called here a ghost string vessel or briefly string ghost. Images obtained from Case 1 (myocardial infarction, NoSVD) (**a**, **c** and **d**), Case 6 (non-hodgkin lymphoma, “pure” SVD) (**b** and **e**), and Case 9 (chronic hypertension, “pure” SVD) (**f**). For viewing tubular and ghost string vessels, also see Additional file 2: Video S1 of Vessels**.** Thin black arrows: vascular bags; thick black arrows: origin or segment of string vessel. Scale bars: 50 μm

In thick sections, we could identify four types (S1-S4) of string vessels with COLL4-immunoreactive walls (Fig. 6). The first type exhibited focal loss of the UEA-l-positive endothelial glycocalyx (S1 in Fig. 6a), but string-like vessel segments with irregularities in the endothelial layer were also observed (Fig. 6b). The second type of string vessels were formed by wide collagenous tubes (S2 in Fig. 6c), and the third type by collapsed tubes (partial constrictions in wide string vessels or fully collapsed, S3 in Fig. 6d, e), whereas the fourth type consisted of discontinuous remnants of string vessels that still connected vessels, but were broken up into individual segments. The latter string vessels were named ghost string vessels, or briefly string ghosts (S4 in Fig. 6f).

### String vessel density is increased in SVD but the overall vessel density is not altered

**Fig. 7:**
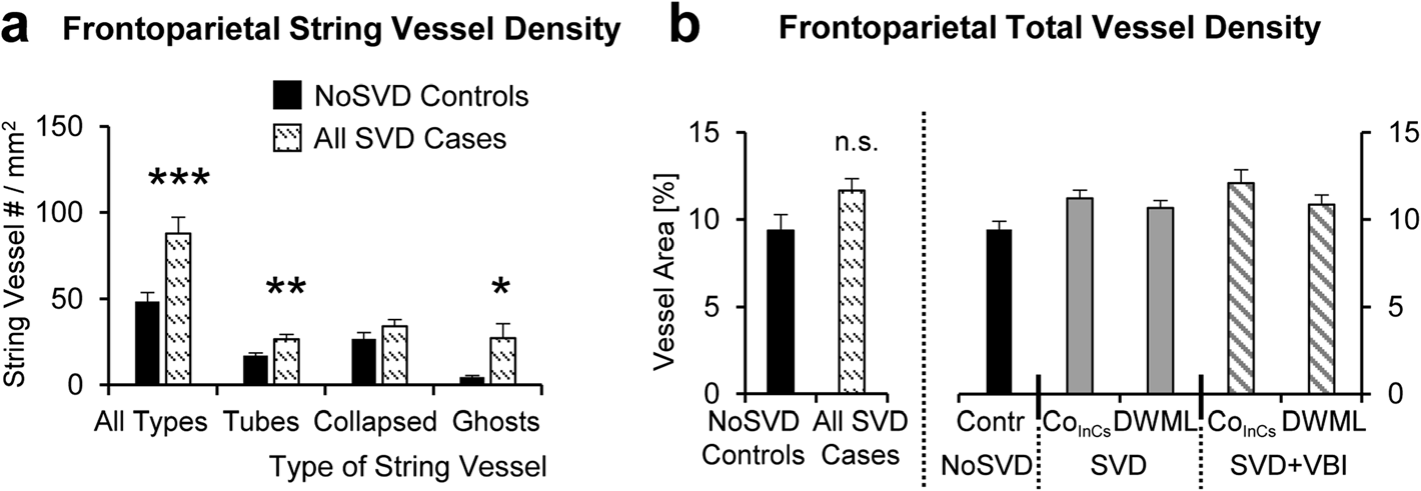
Quantitative analyses of the density of string vessels and of the overall vessel density in the frontoparietal white matter. **a** The density of all types of string vessels as well as the density of string vessel tubes (S2 type) and string vessel ghosts (S4 type) is significantly increased in all SVD cases (pure SVD and SVD + VBI) when compared to NoSVD controls, whereas the density of collapsed/constricted string vessels (S3 type) is not affected (see Fig. 6 for morphology of S2 to S4 type string vessels quantified). **b** In contrast, the overall density of frontoparietal small vessels is not altered when all SVD cases are compared to NoSVD controls (left) or if a more detailed analysis is performed, in which DWMLs are compared to in-case control areas in the two SVD groups or to control areas in NoSVD cases (right). The “pure” SVD cases were referred to as SVD in the diagrams. * *p* < 0.05, ** *p* < 0.01, *** *p* < 0.001, n.s. not significant

After characterizing the morphology of string vessels, the different types of string vessel (S2-S4) were quantified in the frontoparietal white matter except the rare S1 type vessels (Fig. 7a). Two-way ANOVA (vascular disease x presence of DWML) showed that only the total density of string vessels was significantly increased by vascular disease (F_1,24_ = 4.924, *p* = 0.036), but the presence of DWMLs had no effect on the string vessel density, and there was no interaction between the two main factors. Since no differences were detected between pure SVD and SVD + VBI in posthoc comparisons, the two groups were pooled for further analyses. A comparison of string vessel densities between NoSVD controls and all SVD cases (both pure SVD and SVD + VBI) showed a significant increase in SVD for the density of all types of string vessels (*t* = − 3.63, df = 26.8, *p* = 0.001), the S2 string tubes (*t* = − 2.879, df = 26.9, *p* = 0.008) and the S4 string ghosts (*t* = − 2.624, df = 20.4, *p* = 0.016), but not the S3 type collapsed string vessels (*t* = − 1.042, df = 27, n.s.).

Next, we studied whether the overall density of small vessels was altered in SVD (Fig. 7b), but the percentage of white matter area covered by microvessels was not affected by vascular disease (F_2,170_ = 0.043, *p* > 0.10) and presence of DWMLs (F_2,170_ = 1.071, *p* > 0.10), and did not differ between the frontoparietal and temporal white matter (F_2,170_ = 1.663, *p* > 0.10) as shown by three-way ANOVA. In addition, no differences were found in vessel densities when comparing all SVD cases with NoSVD controls using a t-test (*t* = − 1.826, df = 25, *p* = 0.08).

### Density and size of CNS macrophages was significantly increased in SVD

**Fig. 8:**
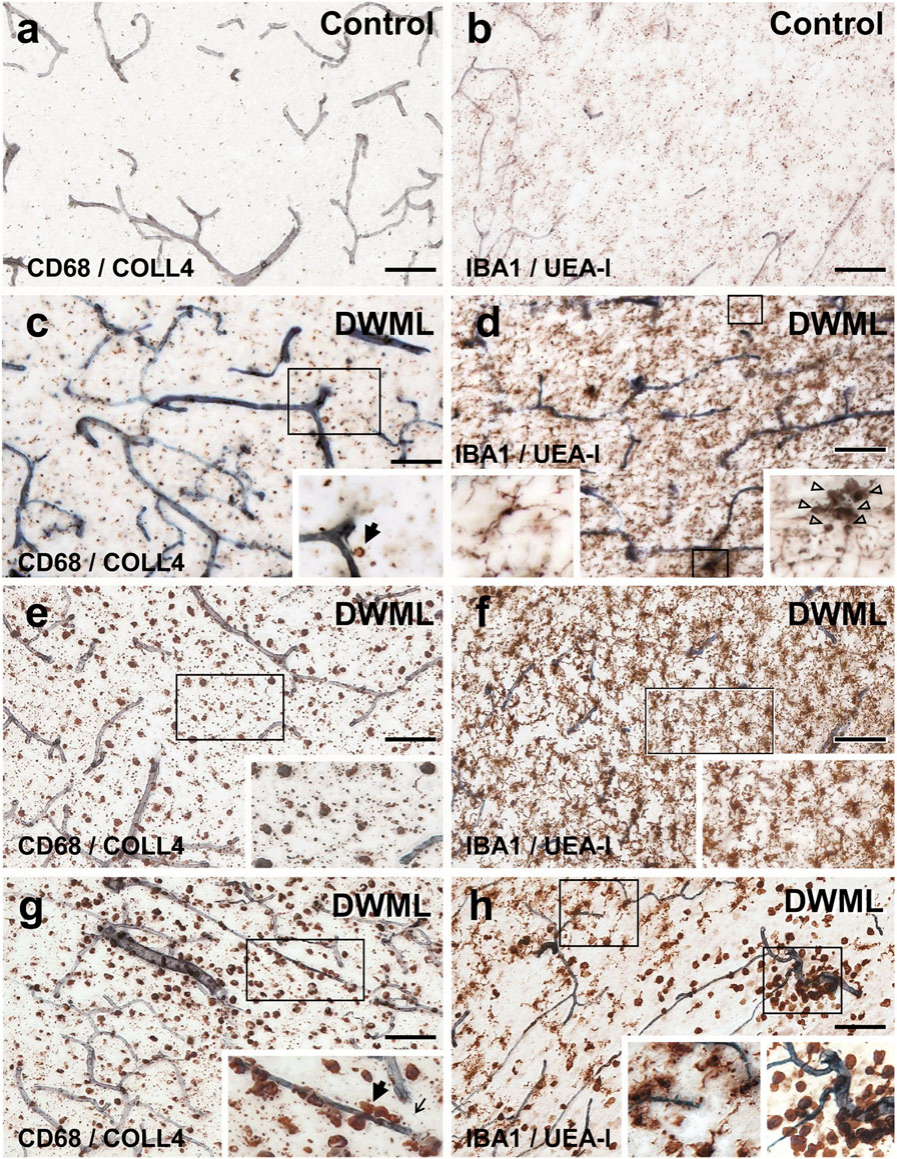
Parenchymal and perivascular CD68-positive macrophages and IBA1-positive microglial cells in white matter areas in thick sections double-labeled for COLL4 and UEA-l, respectively. **11a**-**b** In NoSVD controls, IBA1- and CD68-positive cellular processes are observed in the subcortical white matter, but the density of labeled cell bodies is rather low compared to SVD cases. **c**-**d** In “pure” SVD, more IBA1- and CD68-positive cells with thicker processes are found in the white matter parenchyma compared to NoSVD. Some CD68 positive cells have a close relationship to the wall of vessels. Many IBA1-labeled cells have complex morphologies and clusters with IBA1-labeled cells somata are seen. **e**-**h** In cases with SVD + VBI, DWMLs exhibit numerous enlarged CD68-positive macrophages with a phagocytic phenotype, and COLL4-positive dots are found among CD68-positive cells (thin arrow in inset of **g**). The density of IBA1-positive microglial cells is also high in the DWMLs, and their morphology indicates an activated (hypertrophic) or a phagocytic (round) state. Thick arrows (insets in **c** and **g**) show examples of CD68-positive cells quantified at perivascular sites with a direct association to the wall of COLL4-positive vessels. Images were obtained from Case 2 (ovarian cancer, NoSVD) (**a**-**b**), Case 5 (pulmonary embolism, pure SVD) (**c**-**d**), and Case 12 (pontine bleeding, SVD + VBI) (**e-h**). For a more detailed morphology of IBA1-positive cells, also see Additional file 3: Video S2 of Microglia. Scale bars: 100 μm (**a**, **c**, **e** and **g**) and 50 μm (**b**, **d**, **f** and **h**)

**Fig. 9:**
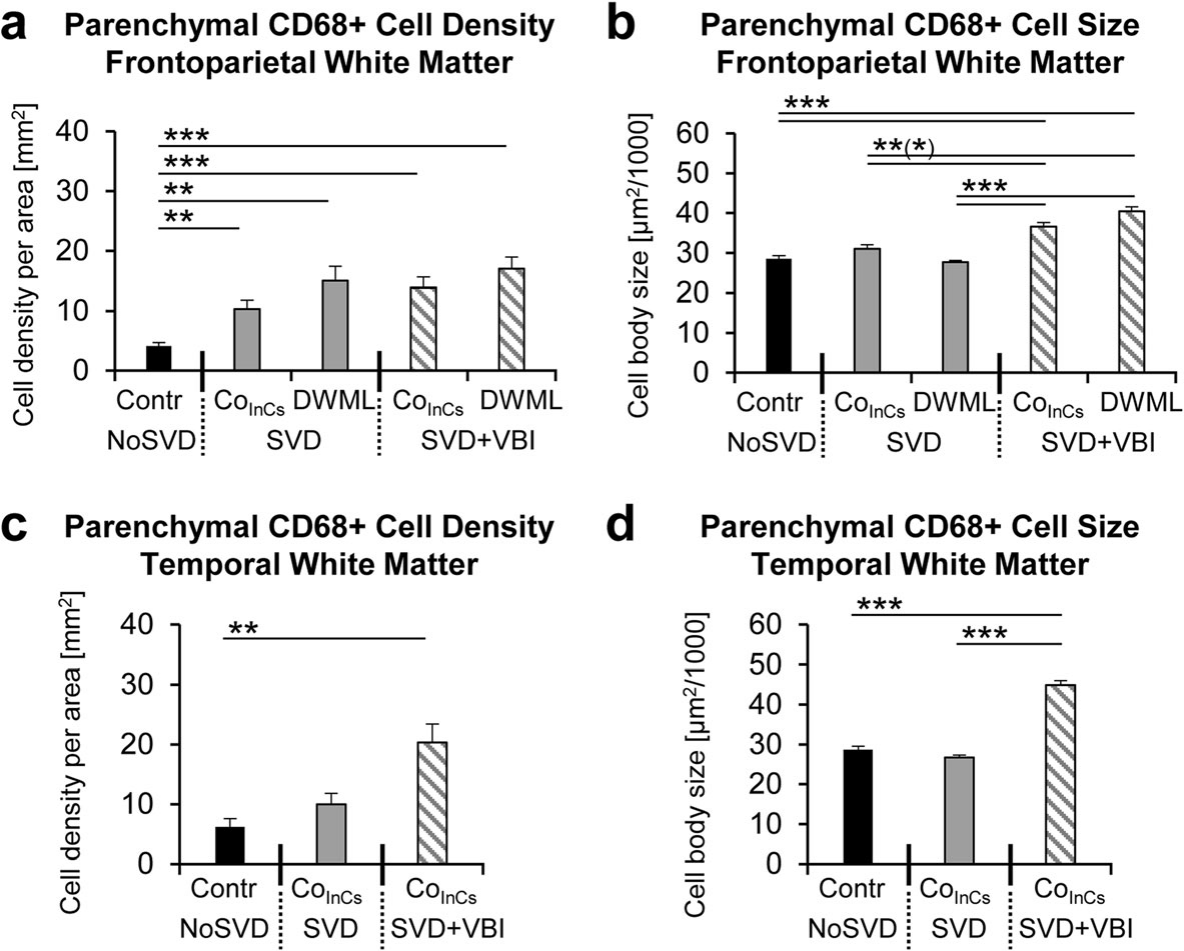
Quantitative analyses of CD68-positive cells. **a**-**b** In pure SVD, the density of parenchymal and perivascular cells is higher in DWMLs compared to the frontoparietal control region, and the same is also true for parenchymal cells quantified in the frontoparietal in-case control area. In SVD associated with VBI, there is a prominent increase in the density of parenchymal and perivascular cells in all white matter areas compared to corresponding control tissue except for perivascular cells in DWMLs. **c**-**d** CD68-positive parenchymal cells are significantly enlarged in all areas in SVD + VBI, whereas these changes are less prominent for perivascular cells. Notably, the size of perivascular cells is also significantly higher in DWMLs of SVD + VBI cases compared to DWMLs in pure SVD. * *p* < 0.05, ** *p* < 0.01, *** *p* < 0.001

White matter regions of NoSVD controls displayed mainly IBA1- and CD68-positive thin cellular processes and relatively few labeled cell bodies. In contrast, IBA1-positive microglial cells were highly branched and had hypertrophic cell bodies in SVD. Round and ameboid cells suggesting a phagocytic phenotype showed both IBA1 and CD68 immunoreactivity and appeared to be more numerous in SVD (Fig. 8). Therefore, CD68-positive cells were quantified in the white matter parenchyma (Fig. 9) and at perivascular sites (see Additional file 7: Figure S4 showing perivascular analyses). Three-way ANOVA (vascular disease x presence of DWML x white matter location) indicated that the density of parenchymal microglial cells was significantly increased by vascular disease F_2,394_ = 9.230, *p* = 0.003) and presence of DWML (F_2,394_ = 11.068, *p* = 0.001), and was also influenced by the white matter location (F_1,394_ = 5.882, *p* = 0.016) and the covariate age (F_1,394_ = 99.371, *p* < 0.001). Posthoc analyses showed an increased density of parenchymal macrophages in SVD (pure or with VBI) in frontoparietal areas (DWMLs and/or in-case control) when compared to NoSVD controls. However, the two SVD groups did not show differences in cell densities between the DWML site and in-case control areas, suggesting a generalized microglial activation).

As the size of macrophages can be an indicator of their functional status, next the cell body area of parenchymal CD68-positive cells was analyzed with three-way ANOVA (Fig. 9; vascular disease x presence of DWML x white matter location). Results indicated that the size of parenchymal CD68-positive cells was significantly increased by vascular disease (F_2,2210_ = 248.516, *p* < 0.001), which showed an interaction with presence of DWMLs (F_1,2210_ = 10.744, *p* = 0.001) and the white matter location (F_1,2210_ = 43.811, *p* < 0.001). Posthoc analyses revealed that parenchymal CD68-positive cells were larger in DWMLs and the temporal white matter in SVD + VBI compared to both pure SVD and/or NoSVD. In addition, the covariate age had a significant effect on the density (F_1,394_ = 99.371, *p* < 0.001) and size (F_2,2210_ = 43.867, *p* < 0.001) of parenchymal microglial cells as indicated by three-way ANOVA (vascular disease x presence of DWML x white matter location).

### CNS macrophages were found in white matter zones with ghost vessels

**Fig. 10:**
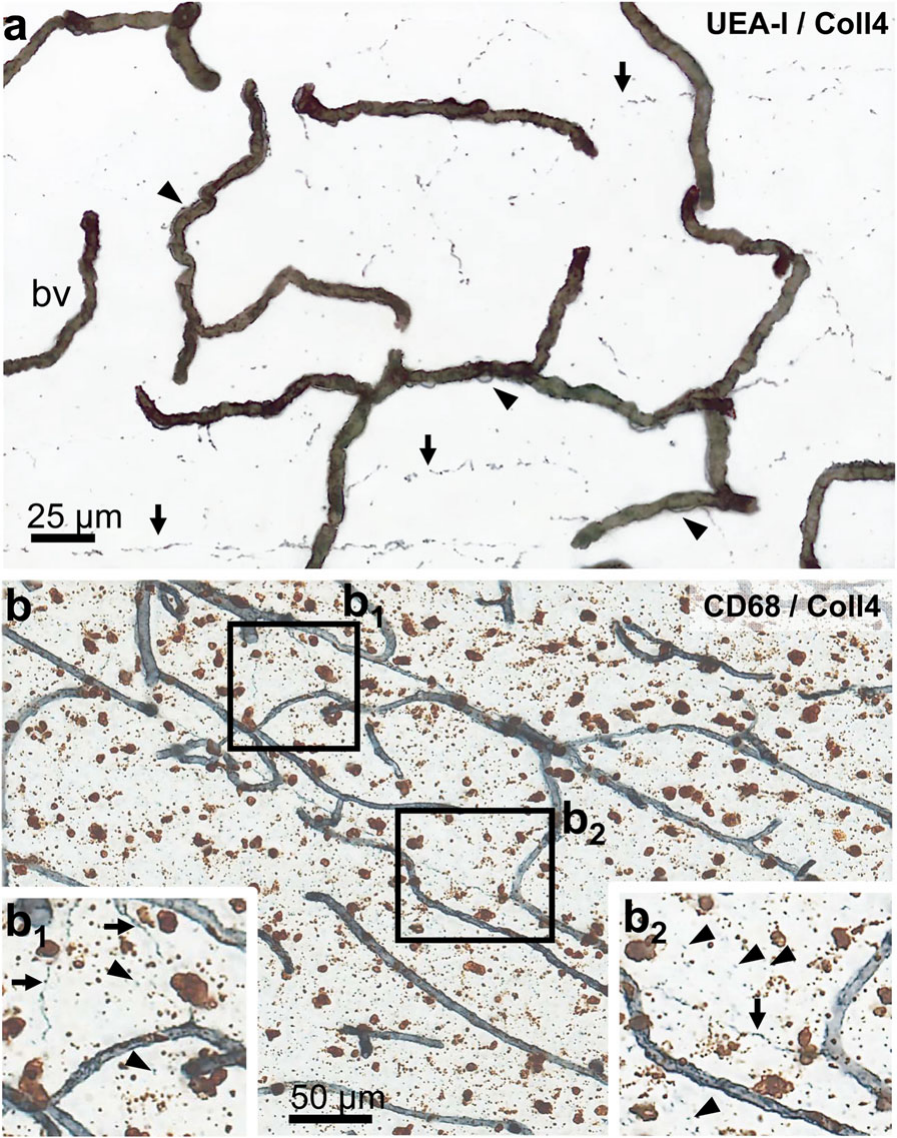
Vessels and CD68-positive cells in z-stack images. **a** Image reconstructed from 57 individual images captured from a single DWML (double-labeling for UEA-l (brown) and COLL4 (blue)) shows what appears to be a network of former string vessels (arrows). Also, numerous vessels with vascular bags are seen (arrow heads) in this area. In our SVD cases, such vascular networks consisted of many ghost vessels and their branches, which had the appearance of beaded threads and dotted lines running over long distances. **b** Sections double-labeled for CD68 (brown) and COLL4 (blue) show accumulation of numerous parenchymal CD68-positive cells in areas with a high density of COLL4-positive ghost vessels (arrows) and dots (arrow heads), suggesting phagocytosis of vessel remnants by CNS macrophages. Images obtained from Case 6 (non-hodgkin lymphoma, pure SVD) (**a**) and Case 12 (pontine bleeding, SVD + VBI) (**b**). For viewing string vessels and COLL4-positive dots, also see Additional file 2: Video S1 of Vessels**.** Scale bars: 25 μm (**a**) and 50 μm (**b**)

To pursue the hypothesis that activated CNS macrophages are involved in the removal of pathologically altered vessels, vessels traveling over large areas were analyzed in z-stack images obtained from 100 μm thick sections. The z-stacks revealed large areas with networks of COLL4-positive ghost vessels in DWMLs (Fig. 10a), which resembled the string ghosts described above (Fig. 6). Further evaluation of the same white matter locations used for quantitative analyses revealed several areas with networks of string ghosts in SVD, some of which were large (*40% of pure SVD cases*: 3 in DWMLs as well as 2 in frontoparietal and 1 temporal in-case control area; *40% of SVD + VBI cases*: 3 DWMLs as well as 2 frontoparietal and 1 temporal in-case control area), whereas in NoSVD controls areas containing a higher density of string ghosts were rather small and often barely detectable (*75% of controls*: 2 frontoparietal and 2 temporal). Moreover, double-label IHC showed a high density of CD68-positive cells with a phagocytic phenotype in white matter areas, which were covered with networks of string ghosts (Fig. 10b).

## Discussion

This study sought to characterize vascular abnormalities and microglial changes in SVD patients. We discovered collagenous pouches and tubes around small vessels, now referred to as vascular bagging, which we suggest to use as a marker of SVD. Key outcomes were the identification of (i) multiple layers of collagenous vascular bags supporting a chronic disease process, (ii) plasma proteins within the vascular bags indicating a porous endothelium and basement membrane, (iii) four types of string vessels suggesting different stages of string vessel formation, and (iv) an enhanced density of COLL4-positive string vessels and ghost vessels that resembled remnants of string vessels. Infiltration of areas with networks of ghost vessels by CD68-positive CNS macrophages further supported the phagocytic removal of vessel fragments. The fact that the overall density of small vessels in the white matter was not altered pointed to a continuous replacement of vessels.

### Generalized white matter involvement and role of aging in SVD

One indication that the changes in our SVD cases are of a chronic nature was the presence of vascular bagging formed by multiple layers of collagen around vessels. Ultrastructural studies have found splitting, branching and thickening of the capillary basement membrane and perivascular deposition of collagen, also called microvascular fibrosis, in the brains of aged rats [24, 40] and rhesus monkeys [51, 67] as well as in aged individuals or Alzheimer’s disease patients [22, 29, 30, 79]. Our current analyses extend these previous observations to vascular bagging in the frontoparietal and temporal control deep white matter.

Quantitative analyses further showed that vascular bagging in SVD is not restricted to small vessels located within the DWMLs, but extends beyond the visible lesions to neighboring frontoparietal white matter areas. Likewise, enhanced densities of activated microglia and CNS macrophages were not confined to the DWMLs consistent with previous observations made in SVD [89], although the prior study included cases with concomitant neurodegenerative pathology. Enlarged perivascular Robin-Virchow spaces also occur in widespread subcortical areas in SVD as shown in previous histopathological investigations [50, 56] and imaging studies, which have confirmed a statistical association between enlarged MRI-visible perivascular spaces and white matter hyperintensities [6, 68].

Therefore, vascular bagging may develop in the frontoparietal and temporal white matter before manifestation of further parenchymal damage, especially in “pure” SVD (without additional VBI), where this pathology was more severe in the DWMLs than in-case control regions. Nevertheless, vascular bagging was comparatively mild in the temporal lobe, compatible with the relative resistance of this region to the development of DWMLs [88]. The progressive character of SVD is also supported by deterioration of astrocytic function at late stages of white matter hyperintensities as shown in a recent clinicopathological study [21]. Similarly, MRI studies provide hints that reversible changes in the normal-appearing white matter such as altered interstitial fluid mobility and water content may precede permanent late-stage changes including demyelination and axonal damage [85]. Altogether these findings support the view that SVD is an age-related generalized and progressive white matter disease fueled by a chronic disease process that globally affects the white matter rather than only the lesion site.

### Vascular bagging is a common feature of SVD with DWMLs

Atrial fibrillation, hypertension and left ventricular hypertrophy are commonly associated with white matter hyperintensities in SVD patients [11, 36, 72, 73]. Studies matching white matter hyperintensities to histological sections have shown that the hyperintensities reflect myelin pallor and dilatation of perivascular spaces and only rarely represent lacunar infarcts [31, 55, 56, 69]. Although subcortical lacunar infarctions are common in patients with WMLs and their presence is a requirement for the diagnosis of Binswanger’s disease [43, 80], there may be some differences in the pathogenesis of diffuse WMLs (leukoaraiosis) and SVD with lacunar infarcts [70, 75]. Especially, Binswanger’s disease and lacunar infarcts have been linked to hypertension [1, 20], which causes atherosclerosis of small arteries and arteriolosclerosis [50, 52, 71]. Moreover, patients with pure lacunar infarctions showed hypercoagulability [77], and an altered haplotype of the endothelial nitric oxide gene [38]. Studies of endothelial dysfunction further showed elevated blood levels of the intercellular adhesion molecule ICAM1 in patients with lacunar infarctions [39], whereas VCAM1 and P-selectin were increased in cases with WMLs at the periventricular region but not at deep subcortical locations [27]. Therefore, further analyses are needed to study the role of our pathological markers of DWMLs/leukoaraiosis in SVD forms such as Binswanger’s disease or hypertensive SVD.

Typical features of hypertensive arteriolosclerosis include hyalinosis/lipohyalinosis and fibrinoid necrosis in arterioles with calibers larger than 40 μm, which is accompanied by deposition of fibrohyaline material and blood-derived lipids in the vessel wall and narrowing of the lumen [52, 64]. The walls of sclerotic medullary arterioles with diameters > 30 μm were also shown to contain multiple layers of collagen I- and IV-positive membranes in various forms of hereditary and sporadic SVD including Binswanger’s disease [23, 53]. However, only one case in our SVD cohort showed prominent hyalinosis of larger arterioles, consistent with previous observations showing manifestation of WMLs in the absence of arteriosclerotic lesions [48]. Furthermore, hypertensive arteriolosclerotic lesions are predominantly located in the basal ganglia [52], whereas this study focused on deep white matter areas. We could demonstrate here that smaller vessels including pre-capillary arterioles and capillaries exhibit vascular bags with COLL4-positive walls in SVD cases with DWMLs, including sporadic disease and SVD associated with remote VBI. Post-capillary venules may also show vascular bagging in SVD, as they cannot be distinguished from capillaries merely based on vessel diameters, deformed erythrocytes squeezing through vessels or the presence of pericytes [7].

### Plasma proteins trapped in vascular bags point to blood brain barrier (BBB) disruption

Accumulation of the plasma proteins FIBR and IgG in vascular bags points to a disruption of the blood brain barrier (BBB) with a porous endothelial cell layer and basement membrane. While circular deposition of FIBR around capillaries was previously reported in the brains of Binswanger’s disease patients, it was concluded that FIBR was located in the perivascular space [3]. Here, we could provide evidence that FIBR and IgG are located within the vascular bags, whereas the widened perivascular space outside the bags did not contain plasma proteins except in areas with leakage to the white matter parenchyma. Plasma protein aggregation in the white matter has been well-established in SVD and is classically regarded as a histopathological indicator for a disrupted BBB [15, 54, 56, 89]. The outbreak of plasma proteins from vascular bags to the surrounding white matter parenchyma further indicates a discontinuity of the outer collagenous membranes of the bags.

Apart from endothelial cells and the basement membrane, astrocytic end feet and pericytes are also required for the structural integrity and maintenance of the BBB [63, 66]. In a clinicopathological study, degeneration of astrocytes with clasmatodendrocytic morphologies and aberrant subcellular expression of aquaporin 4 pointed to a breakage of the BBB in areas with white matter hyperintensities [21]. Loss of pericytes can also lead to a BBB breakdown and exudation of plasma proteins by reducing capillary perfusion as it has been shown in a mouse model of SVD with impaired PDGFR-beta signaling [8, 37]. While the pericytes found here were located outside the vascular bags, these cells and their processes are enclosed by two layers of the basement membrane [67], and therefore their degeneration might contribute to splitting of the basement membrane. The disruption of the BBB in SVD is also supported by in vivo perfusion-weighted MR imaging performed living patients [4].

### Vascular bagging may be triggered by hypoperfusion

Experimental evidence favors the notion that endothelial changes are an early phenomenon of DWMLs [42], and therefore interventions that prevent endothelial damage might have a positive impact by halting SVD progression. Chronic cerebral hypoperfusion has been shown to induce the development of features of SVD including endothelial cell damage, thickening of the capillary basement membrane, gliovascular disruption and WMLs in rodents, which was associated with cognitive deficits [25, 41, 45, 78]. In patients, the impact of reduced cerebral blood flow on the development of leukoaraiosis was demonstrated in a longitudinal MRI study [10]. Glial expression of the hypoxia-inducible factors HIF1α and HIF2α was also elevated in SVD patients with DWMLs [32].

Endothelial damage is the first step in the formation of string vessels [16]. Among the four types of string vessels found here, vessels with endothelial cell recession probably represent early-stage string vessels, whereas wide open or collapsed empty collagenous tubes devoid of an endothelial cell layer correspond to late-stage string vessels. Healthy individuals also have string vessels in the CNS and other organs [16], but in SVD we found an increased density of string vessels and string ghosts that were still connected to vessels or formed extended networks. Especially the higher density of string ghosts found here suggests removal of vessel remnants from the white matter parenchyma in SVD. Alternatively, the empty basement membrane tubes left behind after the death of endothelial cells might guide regrowth of endothelial cells, which can synthetize new layers of basement membrane [49]. Thus, the multi-layered vascular bags found here may be the consequence of a chronic disease process with recurrent recanalization of empty collagenous scaffolds and repeated duplications of the basement membrane.

### Role of microglia in remodeling of the white matter microvasculature

Microglia were visualized with the marker IBA1 that is expressed in both resting and activated microglial cells and phagocytic CNS macrophages were identified with CD68 [83]. Similar to the observations by Young et al. [89], we detected activated IBA1-positive microglial cells and increased cell densities/sizes of CD68-positive macrophages in both DWMLs and the normal appearing white matter in SVD. Moreover, the increase in the density and size of macrophages was more prominent in the SVD groups with VBI compared to pure SVD. Activated microglia with truncated processes and lipid-laden macrophages immunoreactive for HLA-DR as well as clusters of microglia/macrophages labeled for leukocyte common antigen (LCA) were also described in Binswanger’s disease [2]. DWML sites covered with networks of string ghosts were infiltrated by activated microglial cells and CNS macrophages suggesting that they sequestered the damaged vessels. These findings suggest chronic remodeling of vessels in SVD, because despite removal of ghost vessel networks by microglia the overall vessel densities were not altered in our SVD cases.

## Conclusion

Here we report two novel findings in SVD, which are (i) vascular bags filled with leaked plasma proteins and (ii) an enhanced density of string ghosts in white matter areas that were also discovered in areas with high macrophage densities. Under the assumption that endothelial damage is the common driver of both types of lesions, the following scenario could be hypothesized for the chronic disease process in DWMLs, and to some extent, also in the normal appearing white matter in SVD. Endothelial cell damage and loss may result in the formation of empty collagenous tubes and scaffolds that can either lead to string vessel formation or persist, possibly over a long duration. Regrowth of the endothelium into persisting empty basement membrane tubes may induce the synthesis of new layers of collagenous membranes. Age-related changes in the extracellular matrix and degeneration of pericytes may also contribute to the thickening and splitting of basement membranes. This may result in the formation of the multi-layered collagenous vascular bags found in this study and leakage of plasma proteins into the bags or white matter parenchyma. When vessels are not recanalized after endothelial damage, they may eventually collapse leading to formation of various stages of string vessels. Degradation of late-stage string vessels by activated microglial cells may be related to the high density of CD68-positive cells found in areas with networks of string ghosts and their remnants.

## Additional files

Additional file 1: Patient data. Demographic data and neuropathological diagnoses are shown for cases with small vessel disease (SVD) with/without vascular brain injury (VBI) and NoSVD controls included to the study. (DOCX 19.1 kb)

Additional file 2: Video of vascular bags and string vessels. The video was constructed from 116 images taken with the 20x objective at equidistant 0.7 μm steps throughout the section. Vessels are double-labeled for collagen IV (COLL4) and lectin (UEA-l) using (immuno)histochemistry. Vessel pathology is shown in the deep white matter lesion (DWML) of a case with SVD and a contralateral subacute ischemic cerebral infarct (Case 14). Vascular bags formed by collagenous membranes around vessels are attached to the vessel walls. A string vessel with a short constricted segment becomes discontinuous. Therefore, the latter types of vessel remnants were called ghost string vessels or briefly string ghosts. In addition, an empty collagenous tube without an endothelial layer is seen that may correspond to an early non-collapsed string vessel. Please also note the multiple COLL4-positive dots in the white matter parenchyma, which form vessel-like shaped rows in z-stack images. (AVI 16324 kb)

Additional file 3: Video of IBA1-positive microglia. The video was constructed from 100 images taken with the 20x objective at equidistant 0.7 μm steps throughout the section. The section double-labeled for the IBA1 and lectin shows complex microglia morphologies in the in-case control region of a SVD case with an ischemic cerebral infarct in the contralateral internal capsule (Case 10). (AVI 16671 kb)

Additional file 4: White matter spongiosis in SVD. Images show immunohistochemical demonstration of myelin basic protein (MBP) and myelin staining performed with a modified Heidenhain procedure. **a** and **c**: Deep white matter lesions (DWMLs) show a loosening of the white matter resulting in spongiosis and a crisscross pattern of remaining axons due to loss of MBP-positive/myelinated fiber tracts (Case 6, SVD). **b** and **d**: In contrast, the control white matter (Case 2, NoSVD) contains well-organized fiber tracts with bundles of longitudinal axons (LAx) and horizontal axons (HAx). Scale bars: 50 μm. (TIF 19818 kb)

Additional file 5: Drawing of vascular bagging. The drawing gives an overview of the vessel types and their proportion (%) among all vessel segments analyzed. Brown color represents labeling of the endothelium with UEA-l and blue color COLL4-positive membranes. Type 1 vessels with an intact endothelium and basement membrane are the most common type in the study population, whereas type 2 vessels with irregularities restricted to the endothelium are rare. DWMLs express a high proportion of type 3 vessels with vascular bags. Likewise type 4 vessels with changes of both the endothelial cell layer and external collagenous membranes are common. (TIF 12345 kb)

Additional file 6: Distribution of diameters. The diagram indicates the distribution of the diameters of small vessels (vessel calibers) (**a**) and maximum diameters at the outer membrane of the vascular bags (**b**). The diameters reported were determined in vessel segments that were in focus in images taken with the 20x objective and were used for quantitative analyses of vascular bags (see also Fig. 2). The width of the vascular bags was calculated as the difference between the two diameters (see also Fig. 3). (TIF 19068 kb)

Additional file 7: Diagram of perivascular CD68-positive macrophages showing the density (**a** and **c**) and size (**b** and **d**) of cells in the frontoparietal (**a-b**) and temporal white matter (**c-d**) analyzed with three-way ANOVA (vascular disease x presence of DWML x white matter location) and the posthoc Games-Howell test. In the three-way ANOVA, the density of perivascular macrophages was significantly increased by vascular disease (F_2,394_ = 8.479, *p* = 0.004) and presence of DWMLs (F_2,394_ = 11.665, *p* = 0.001), and also depended on the white matter location (F_1,394_ = 9.135, *p* = 0.003). Moreover, the size of perivascular CD68-positive cells was significantly affected by vascular disease (F_2,2856_ = 65.003, *p* < 0.001) in interaction with the white matter location (F_1,2856_ = 19.668, *p* < 0.001), and the covariate age had a significant effect on the density (F_1,394_ = 65.231, *p* < 0.001) and size (F_2,2856_ = 9.527, *p* = 0.002) of the cells. **a-b:** In the frontoparietal white matter, posthoc analyses revealed a higher density of perivascular macrophages in DWMLs of all SVD cases (pure SVD, SVD + VBI) compared to NoSVD. Also, CD68-positive cells were significantly enlarged in the DWMLs of SVD + VBI cases compared to DWMLs in pure SVD. Neither the density nor the size of CD68-positive cells was significantly altered in the in-case control areas of SVD cases compared to the control white matter in NoSVD cases. **c-d:** In the temporal white matter, posthoc analyses indicated that the density of CD68-positive cells was significantly increased in-case control areas of SVD + VBI cases but not of pure SVD cases when compared to NoSVD controls. Notably, perivascular cells were significantly larger in DWMLs of SVD + VBI cases than in DWMLs of pure SVD cases or in the control white matter of NoSVD cases. The density or size of CD68-positive cells in in-case control areas in pure SVD was not altered when compared to NoSVD. * *p* < 0.05, ** *p* < 0.01, *** *p* < 0.001. (TIF 19710 kb)

## References

[1] Akiguchi I, Budka H, Shirakashi Y, Woehrer A, Watanabe T, al SA (2014). MRI features of Binswanger's disease predict prognosis and associated pathology. Ann Clin Transl Neurol.

[2] Akiguchi I, Tomimoto H, Suenaga T, Wakita H, Budka H (1997). Alterations in glia and axons in the brains of Binswanger’s disease patients. Stroke.

[3] Akiguchi I, Tomimoto H, Suenaga T, Wakita H, Budka H (1998). Blood-brain barrier dysfunction in Binswanger’s disease; an immunohistochemical study. Acta Neuropathol.

[4] Arba F, Leigh R, Inzitari D, Warach SJ, Luby M, Lees KR (2017). Blood-brain barrier leakage increases with small vessel disease in acute ischemic stroke. Neurology.

[5] Aribisala BS, Valdes Hernandez MC, Royle NA, Morris Z, Munoz Maniega S, Bastin ME (2013). Brain atrophy associations with white matter lesions in the ageing brain: the Lothian birth cohort 1936. Eur Radiol.

[6] Aribisala BS, Wiseman S, Morris Z, Valdes-Hernandez MC, Royle NA, Maniega SM (2014). Circulating inflammatory markers are associated with magnetic resonance imaging-visible perivascular spaces but not directly with white matter hyperintensities. Stroke.

[7] Attwell D, Mishra A, Hall CN, O'Farrell FM, Dalkara T (2016). What is a pericyte?. J Cereb Blood Flow Metab.

[8] Bell RD, Winkler EA, Sagare AP, Singh I, LaRue B, Deane R (2010). Pericytes control key neurovascular functions and neuronal phenotype in the adult brain and during brain aging. Neuron.

[9] Benakis C, Garcia-Bonilla L, Iadecola C, Anrather J (2014). The role of microglia and myeloid immune cells in acute cerebral ischemia. Front Cell Neurosci.

[10] Bernbaum M, Menon BK, Fick G, Smith EE, Goyal M, Frayne R (2015). Reduced blood flow in normal white matter predicts development of leukoaraiosis. J Cereb Blood Flow Metab.

[11] Birdsill AC, Koscik RL, Jonaitis EM, Johnson SC, Okonkwo OC, Hermann BP (2014). Regional white matter hyperintensities: aging, Alzheimer’s disease risk, and cognitive function. Neurobiol Aging.

[12] Braak H (1976). On the striate area of the human isocortex. A Golgi- and pigmentarchitectonic study. J Comp Neurol.

[13] Braak H, Braak E (1991). Neuropathological stageing of Alzheimer-related changes. Acta Neuropathol.

[14] Braak H, Del Tredici K, Rub U, de Vos RA, Jansen Steur EN, Braak E (2003). Staging of brain pathology related to sporadic Parkinson’s disease. Neurobiol Aging.

[15] Bridges LR, Andoh J, Lawrence AJ, Khoong CH, Poon WW, Esiri MM (2014). Blood-brain barrier dysfunction and cerebral small vessel disease (arteriolosclerosis) in brains of older people. J Neuropathol Exp Neurol.

[16] Brown WR (2010). A review of string vessels or collapsed, empty basement membrane tubes. J Alzheimers Dis.

[17] Brown WR, Moody DM, Challa VR, Thore CR, Anstrom JA (2002). Venous collagenosis and arteriolar tortuosity in leukoaraiosis. J Neurol Sci.

[18] Brown WR, Moody DM, Thore CR, Anstrom JA, Challa VR (2009). Microvascular changes in the white mater in dementia. J Neurol Sci.

[19] Brown WR, Thore CR (2011). Review: cerebral microvascular pathology in ageing and neurodegeneration. Neuropathol Appl Neurobiol.

[20] Caplan LR (2015). Lacunar infarction and small vessel disease: pathology and pathophysiology. J Stroke.

[21] Chen A, Akinyemi RO, Hase Y, Firbank MJ, Ndung’u MN, Foster V (2016). Frontal white matter hyperintensities, clasmatodendrosis and gliovascular abnormalities in ageing and post-stroke dementia. Brain.

[22] Claudio L (1996). Ultrastructural features of the blood-brain barrier in biopsy tissue from Alzheimer’s disease patients. Acta Neuropathol.

[23] Craggs LJ, Hagel C, Kuhlenbaeumer G, Borjesson-Hanson A, Andersen O, Viitanen M (2013). Quantitative vascular pathology and phenotyping familial and sporadic cerebral small vessel diseases. Brain Pathol.

[24] de Jong GI, de Weerd H, Schuurman T, Traber J, Luiten PG (1990). Microvascular changes in aged rat forebrain. Effects of chronic nimodipine treatment. Neurobiol Aging.

[25] De Jong GI, Farkas E, Stienstra CM, Plass JR, Keijser JN, de la Torre JC (1999). Cerebral hypoperfusion yields capillary damage in the hippocampal CA1 area that correlates with spatial memory impairment. Neuroscience.

[26] de Laat KF, Tuladhar AM, van Norden AG, Norris DG, Zwiers MP, de Leeuw FE (2011). Loss of white matter integrity is associated with gait disorders in cerebral small vessel disease. Brain.

[27] de Leeuw FE, de Kleine M, Frijns CJ, Fijnheer R, van Gijn J, Kappelle LJ (2002). Endothelial cell activation is associated with cerebral white matter lesions in patients with cerebrovascular disease. Ann N Y Acad Sci.

[28] Debette S, Markus HS (2010). The clinical importance of white matter hyperintensities on brain magnetic resonance imaging: systematic review and meta-analysis. BMJ.

[29] Farkas E, de Vos RA, Donka G, Jansen Steur EN, Mihaly A, Luiten PG (2006). Age-related microvascular degeneration in the human cerebral periventricular white matter. Acta Neuropathol.

[30] Farkas E, Luiten PG (2001). Cerebral microvascular pathology in aging and Alzheimer’s disease. Prog Neurobiol.

[31] Fernando MS, O'Brien JT, Perry RH, English P, Forster G, McMeekin W (2004). Comparison of the pathology of cerebral white matter with post-mortem magnetic resonance imaging (MRI) in the elderly brain. Neuropathol Appl Neurobiol.

[32] Fernando MS, Simpson JE, Matthews F, Brayne C, Lewis CE, Barber R (2006). White matter lesions in an unselected cohort of the elderly: molecular pathology suggests origin from chronic hypoperfusion injury. Stroke.

[33] Garde E, Lykke Mortensen E, Rostrup E, Paulson OB (2005). Decline in intelligence is associated with progression in white matter hyperintensity volume. J Neurol Neurosurg Psychiatry.

[34] Garde E, Mortensen EL, Krabbe K, Rostrup E, Larsson HB (2000). Relation between age-related decline in intelligence and cerebral white-matter hyperintensities in healthy octogenarians: a longitudinal study. Lancet.

[35] Griffanti L, Jenkinson M, Suri S, Zsoldos E, Mahmood A, Filippini N (2018). Classification and characterization of periventricular and deep white matter hyperintensities on MRI: A study in older adults. Neuroimage.

[36] Hajjar I, Quach L, Yang F, Chaves PH, Newman AB, Mukamal K (2011). Hypertension, white matter hyperintensities, and concurrent impairments in mobility, cognition, and mood: the Cardiovascular Health Study. Circulation.

[37] Hall CN, Reynell C, Gesslein B, Hamilton NB, Mishra A, Sutherland BA (2014). Capillary pericytes regulate cerebral blood flow in health and disease. Nature.

[38] Hassan A, Gormley K, O’Sullivan M, Knight J, Sham P, Vallance P (2004). Endothelial nitric oxide gene haplotypes and risk of cerebral small-vessel disease. Stroke.

[39] Hassan A, Hunt BJ, O’Sullivan M, Parmar K, Bamford JM, Briley D (2003). Markers of endothelial dysfunction in lacunar infarction and ischaemic leukoaraiosis. Brain.

[40] Hicks P, Rolsten C, Brizzee D, Samorajski T (1983). Age-related changes in rat brain capillaries. Neurobiol Aging.

[41] Holland PR, Searcy JL, Salvadores N, Scullion G, Chen G, Lawson G (2015). Gliovascular disruption and cognitive deficits in a mouse model with features of small vessel disease. J Cereb Blood Flow Metab.

[42] Huang Y, Zhang W, Lin L, Feng J, Chen F, Wei W (2010). Is endothelial dysfunction of cerebral small vessel responsible for white matter lesions after chronic cerebral hypoperfusion in rats?. J Neurol Sci.

[43] Huisa BN, Rosenberg GA (2014). Binswanger’s disease: toward a diagnosis agreement and therapeutic approach. Expert Rev Neurother.

[44] Hutchins B, Weber JT (1983). A rapid myelin stain for frozen sections: modification of the Heidenhain procedure. J Neurosci Methods.

[45] Ihara M, Taguchi A, Maki T, Washida K, Tomimoto H (2014). A mouse model of chronic cerebral hypoperfusion characterizing features of vascular cognitive impairment. Methods Mol Biol.

[46] Imaizumi T, Inamura S, Nomura T (2014). The severities of white matter lesions possibly influence the recurrences of several stroke types. J Stroke Cerebrovasc Dis.

[47] Inzitari D, Cadelo M, Marranci ML, Pracucci G, Pantoni L (1997). Vascular deaths in elderly neurological patients with leukoaraiosis. J Neurol Neurosurg Psychiatry.

[48] Inzitari D, Mascalchi M, Giordano GP, Marini P, Sita D, Abbamondi AL (1989). Histopathological correlates of leuko-araiosis in patients with ischemic stroke. Eur Neurol.

[49] Irvine AR, Wood IS (1987). Radiation retinopathy as an experimental model for ischemic proliferative retinopathy and rubeosis iridis. Am J Ophthalmol.

[50] Kalaria RN, Kenny RA, Ballard CG, Perry R, Ince P, Polvikoski T (2004). Towards defining the neuropathological substrates of vascular dementia. J Neurol Sci.

[51] Keuker JI, Luiten PG, Fuchs E (2000). Capillary changes in hippocampal CA1 and CA3 areas of the aging rhesus monkey. Acta Neuropathol.

[52] Lammie GA (2002). Hypertensive cerebral small vessel disease and stroke. Brain Pathol.

[53] Lin JX, Tomimoto H, Akiguchi I, Matsuo A, Wakita H, Shibasaki H (2000). Vascular cell components of the medullary arteries in Binswanger’s disease brains: a morphometric and immunoelectron microscopic study. Stroke.

[54] Ma KC, Lundberg PO, Lilja A, Olsson Y (1992). Binswanger’s disease in the absence of chronic arterial hypertension. A case report with clinical, radiological and immunohistochemical observations on intracerebral blood vessels. Acta Neuropathol.

[55] Matsusue E, Sugihara S, Fujii S, Ohama E, Kinoshita T, Ogawa T (2006). White matter changes in elderly people: MR-pathologic correlations. Magn Reson Med Sci.

[56] McAleese KE, Alafuzoff I, Charidimou A, De Reuck J, Grinberg LT, Hainsworth AH (2016). Post-mortem assessment in vascular dementia: advances and aspirations. BMC Med.

[57] Moody DM, Bell MA, Challa VR (1990). Features of the cerebral vascular pattern that predict vulnerability to perfusion or oxygenation deficiency: an anatomic study. AJNR Am J Neuroradiol.

[58] Moody DM, Brown WR, Challa VR, Ghazi-Birry HS, Reboussin DM (1997). Cerebral microvascular alterations in aging, leukoaraiosis, and Alzheimer’s disease. Ann N Y Acad Sci.

[59] Moody DM, Thore CR, Anstrom JA, Challa VR, Langefeld CD, Brown WR (2004). Quantification of afferent vessels shows reduced brain vascular density in subjects with leukoaraiosis. Radiology.

[60] Mulisch M, Welsch U (2015). Romeis - Mikroskopische Technik.

[61] Munoz DG, Hastak SM, Harper B, Lee D, Hachinski VC (1993). Pathologic correlates of increased signals of the centrum ovale on magnetic resonance imaging. Arch Neurol.

[62] Nyquist PA, Bilgel M, Gottesman R, Yanek LR, Moy TF, Becker LC (2015). Age differences in periventricular and deep white matter lesions. Neurobiol Aging.

[63] Ostergaard L, Engedal TS, Moreton F, Hansen MB, Wardlaw JM, Dalkara T (2016). Cerebral small vessel disease: capillary pathways to stroke and cognitive decline. J Cereb Blood Flow Metab.

[64] Pantoni L (2010). Cerebral small vessel disease: from pathogenesis and clinical characteristics to therapeutic challenges. Lancet Neurol.

[65] Pantoni L, Garcia JH (1997). Pathogenesis of leukoaraiosis: a review. Stroke.

[66] Persidsky Y, Ramirez SH, Haorah J, Kanmogne GD (2006). Blood-brain barrier: structural components and function under physiologic and pathologic conditions. J NeuroImmune Pharmacol.

[67] Peters A, Sethares C (2012). Age-related changes in the morphology of cerebral capillaries do not correlate with cognitive decline. J Comp Neurol.

[68] Potter GM, Doubal FN, Jackson CA, Chappell FM, Sudlow CL, Dennis MS (2015). Enlarged perivascular spaces and cerebral small vessel disease. Int J Stroke.

[69] Revesz T, Hawkins CP, du Boulay EP, Barnard RO, McDonald WI (1989). Pathological findings correlated with magnetic resonance imaging in subcortical arteriosclerotic encephalopathy (Binswanger's disease). J Neurol Neurosurg Psychiatry.

[70] Rosenberg GA, Wallin A, Wardlaw JM, Markus HS, Montaner J, Wolfson L (2016). Consensus statement for diagnosis of subcortical small vessel disease. J Cereb Blood Flow Metab.

[71] Rosenblum WI (2008). Fibrinoid necrosis of small brain arteries and arterioles and miliary aneurysms as causes of hypertensive hemorrhage: a critical reappraisal. Acta Neuropathol.

[72] Rostrup E, Gouw AA, Vrenken H, van Straaten EC, Ropele S, Pantoni L (2012). The spatial distribution of age-related white matter changes as a function of vascular risk factors--results from the LADIS study. Neuroimage.

[73] Ryu WS, Woo SH, Schellingerhout D, Chung MK, Kim CK, Jang MU (2014). Grading and interpretation of white matter hyperintensities using statistical maps. Stroke.

[74] Schmidt R, Seiler S, Loitfelder M (2016). Longitudinal change of small-vessel disease-related brain abnormalities. J Cereb Blood Flow Metab.

[75] Shi Y, Wardlaw JM (2016). Update on cerebral small vessel disease: a dynamic whole-brain disease. Stroke Vasc Neurol.

[76] Thal DR, Rub U, Orantes M, Braak H (2002). Phases of a beta-deposition in the human brain and its relevance for the development of AD. Neurology.

[77] Tomimoto H, Akiguchi I, Ohtani R, Yagi H, Kanda M, Shibasaki H (2001). The coagulation-fibrinolysis system in patients with leukoaraiosis and Binswanger disease. Arch Neurol.

[78] Ueno M, Tomimoto H, Akiguchi I, Wakita H, Sakamoto H (2002). Blood-brain barrier disruption in white matter lesions in a rat model of chronic cerebral hypoperfusion. J Cereb Blood Flow Metab.

[79] Uspenskaia O, Liebetrau M, Herms J, Danek A, Hamann GF (2004). Aging is associated with increased collagen type IV accumulation in the basal lamina of human cerebral microvessels. BMC Neurosci.

[80] van der Flier WM, van Straaten EC, Barkhof F, Verdelho A, Madureira S, Pantoni L (2005). Small vessel disease and general cognitive function in nondisabled elderly: the LADIS study. Stroke.

[81] van der Holst HM, Tuladhar AM, Zerbi V, van Uden IWM, de Laat KF, van Leijsen EMC (2018). White matter changes and gait decline in cerebral small vessel disease. Neuroimage Clin.

[82] Vermeer SE, Hollander M, van Dijk EJ, Hofman A, Koudstaal PJ, Breteler MM (2003). Silent brain infarcts and white matter lesions increase stroke risk in the general population: the Rotterdam Scan Study. Stroke.

[83] Walker DG, Lue LF (2015). Immune phenotypes of microglia in human neurodegenerative disease: challenges to detecting microglial polarization in human brains. Alzheimers Res Ther.

[84] Wardlaw JM, Sandercock PA, Dennis MS, Starr J (2003). Is breakdown of the blood-brain barrier responsible for lacunar stroke, leukoaraiosis, and dementia?. Stroke.

[85] Wardlaw JM, Valdes Hernandez MC, Munoz-Maniega S (2015). What are white matter hyperintensities made of? Relevance to vascular cognitive impairment. J Am Heart Assoc.

[86] Yang P, Pavlovic D, Waldvogel H, Dragunow M, Synek B, Turner C (2015). String vessel formation is increased in the brain of Parkinson disease. J Parkinsons Dis.

[87] Ylikoski A, Erkinjuntti T, Raininko R, Sarna S, Sulkava R, Tilvis R (1995). White matter hyperintensities on MRI in the neurologically nondiseased elderly. Analysis of cohorts of consecutive subjects aged 55 to 85 years living at home. Stroke.

[88] Yoshita M, Fletcher E, Harvey D, Ortega M, Martinez O, Mungas DM (2006). Extent and distribution of white matter hyperintensities in normal aging, MCI, and AD. Neurology.

[89] Young VG, Halliday GM, Kril JJ (2008). Neuropathologic correlates of white matter hyperintensities. Neurology.

