# Supplementary material for: Endothelial damage, vascular bagging and remodeling of the microvascular bed in human microangiopathy with deep white matter lesions
Source: Acta Neuropathol Commun. 2018 Nov 23;6:128. doi: 10.1186/s40478-018-0632-z (PMC6260986; doi:10.1186/s40478-018-0632-z)
Supplement: Supplementary file 1 — Patient data. Demographic data and neuropathological diagnoses are shown for cases with small vessel disease (SVD) with/without vascular brain injury (VBI) and NoSVD controls included to the study. (DOCX 19.1 kb) [file 40478_2018_632_MOESM1_ESM.docx]

| **Patients** | | | | **Demographics and Neuropathology** | |
| --- | --- | --- | --- | --- | --- |
| Age of all cases at death (yrs) | | | | 64.1 (SD = 10.2), male / female n = 7 / n =7 | |
| Age of Control cases (no DWML) | | | | 61.8 (SD = 7.7 ), n = 4 | |
| Age of SVD-only cases without VBI | | | | 63.0 (SD = 13.9), n = 5 | |
| Age of SVD cases with VBI | | | | 67.2 (SD = 9.0), n = 5 | |
| Any neurofibrillary pathology | | | | n = 10 | |
| Neurofibrillary tangle stage | | | | ≤ Stage II n = 14  (Stage 0 n = 4, Stage I n = 9; Stage II n = 1) | |
| Beta-amyloid phases | | | | ≤ Phase II n = 14  (Phase 0 n=6; Phase I n=4; Phase II n=4) | |
| Any parenchymal amyloid | | | | n = 8 | |
| CAA present (few, diffuse) | | | | n = 1 | |
| LB pathology | | | | n = 0 | |
|  | | | | | |
| **Abbreviations:** DWML deep white matter lesion, CAA cerebral amyloid angiopathy,  LB Lewy body, SD standard deviation, SVD small vessel disease, VBI vascular brain injury | | | | | |
| **Notes:**  All SVD cases showed DWML in frontoparietal areas.  VBI corresponded to remote acute/subacute (4 cases) and chronic infarcts (1 case).  Several SVD+VBI cases had subcortical microbleeds (4 out of 5 cases).  One SVD+VBI case had a subcortical lacunar infarct (thalamic).  One pure SVD case (no VBI) suffered from hypertension.  Parenchymal amyloid was located in cerebral cortex/amygdala and was diffuse in 4 out of 8 cases. | | | | | |
| **Case** | **Disease Group** | **Age** | **Sex** | | **Diagnosis** |
| 1 | Control | 65 | m | | Myocardial infarction |
| 2 | Control | 54 | f | | Ovarian cancer |
| 3 | Control | 57 | f | | Esophagus cancer |
| 4 | Control | 71 | f | | Left heart failure with pulmonary edema |
| 5 | SVD | 77 | m | | Pulmonary embolism |
| 6 | SVD | 57 | f | | Highly malignant non-hodgkin lymphoma |
| 7 | SVD | 50 | m | | Renal cancer |
| 8 | SVD | 52 | m | | Gastrointestinal infarct, occlusion of pelvic artery, advanced peripheral artery disease at Fontaine stage IIb to III |
| 9 | SVD | 79 | m | | Chronic hypertension, aortic valve replacement |
| 10 | SVD+VBI | 57 | f | | Breast cancer, ischemic stroke in contralateral internal capsule |
| 11 | SVD+VBI | 67 | m | | Chronic ischemic cerebral infarct (2 yrs old), sudden cardiovascular arrest |
| 12 | SVD+VBI | 69 | f | | Pontine bleeding |
| 13 | SVD+VBI | 62 | m | | Contralateral (sub)acute ischemic stroke (MCA-l) and thalamic infarct |
| 14 | SVD+VBI | 81 | f | | Contralateral (sub)acute ischemic infarction (MCA-l) |
