# Supplementary figures and images for: Endothelial damage, vascular bagging and remodeling of the microvascular bed in human microangiopathy with deep white matter lesions
Source: Acta Neuropathol Commun. 2018 Nov 23;6:128. doi: 10.1186/s40478-018-0632-z (PMC6260986; doi:10.1186/s40478-018-0632-z)

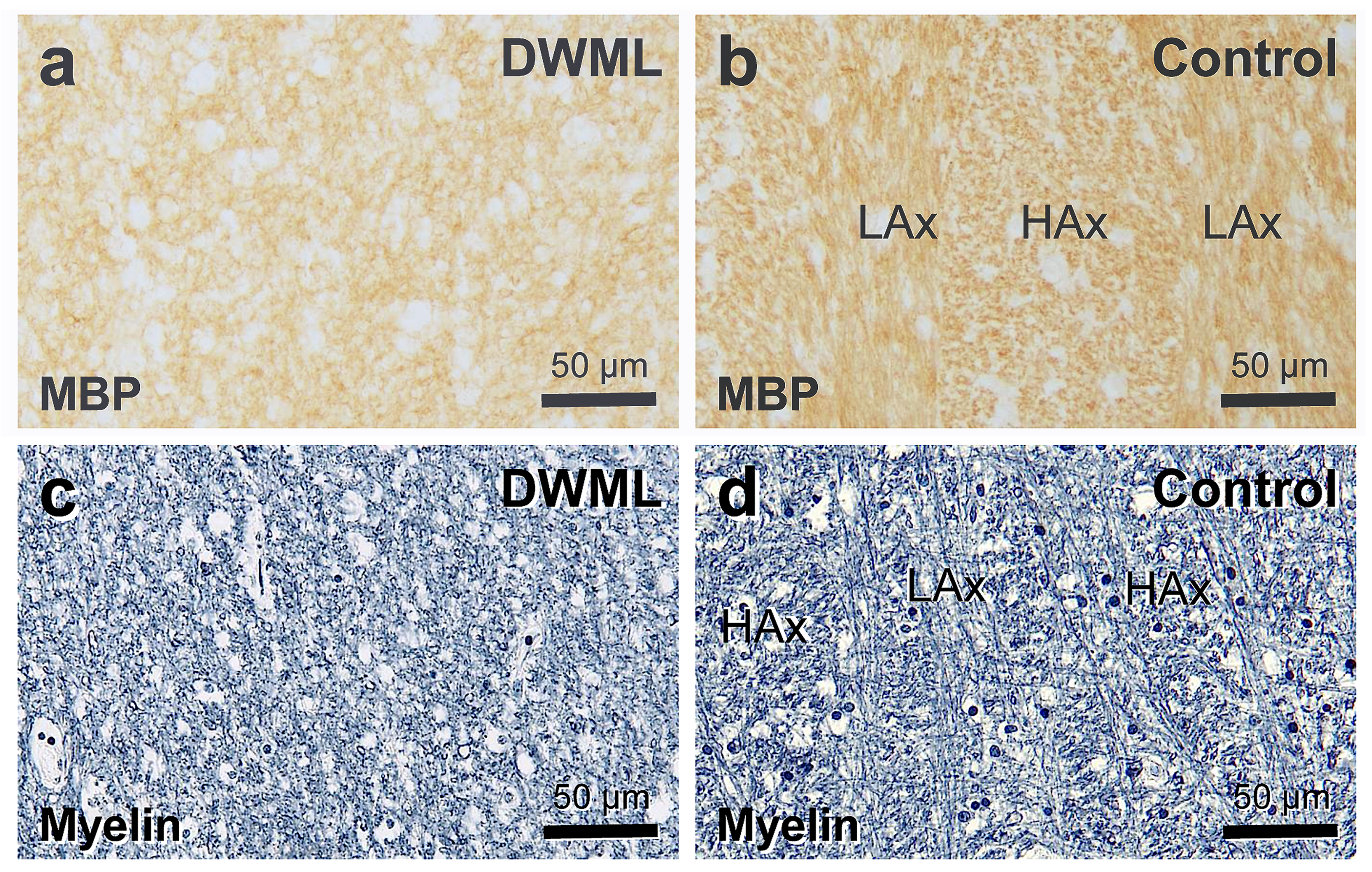

Supplement: Supplementary file 4 — White matter spongiosis in SVD. Images show immunohistochemical demonstration of myelin basic protein (MBP) and myelin staining performed with a modified Heidenhain procedure. a and c: Deep white matter lesions (DWMLs) show a loosening of the white matter resulting in spongiosis and a crisscross pattern of remaining axons due to loss of MBP-positive/myelinated fiber tracts (Case 6, SVD). b and d: In contrast, the control white matter (Case 2, NoSVD) contains well-organized fiber tracts with bundles of longitudinal axons (LAx) and horizontal axons (HAx). Scale bars: 50 μm. (TIF 19818 kb) [file 40478_2018_632_MOESM4_ESM.tif]

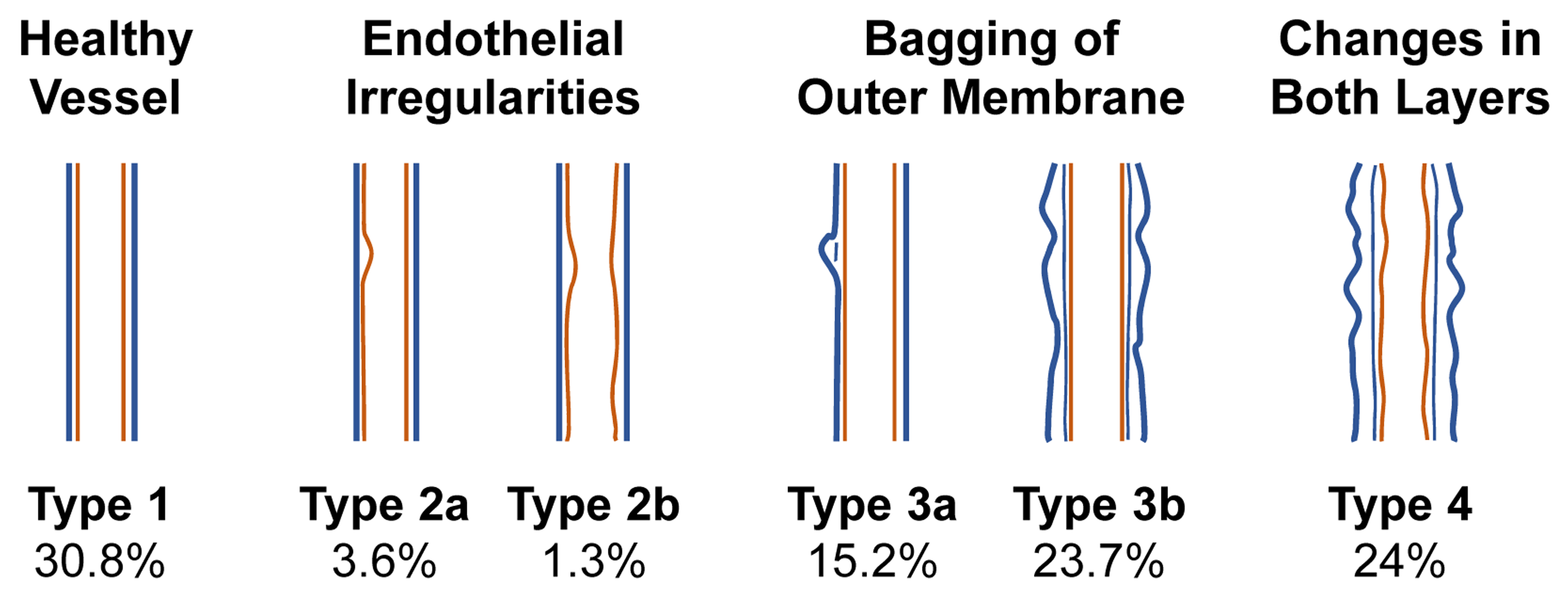

Supplement: Supplementary file 5 — Drawing of vascular bagging. The drawing gives an overview of the vessel types and their proportion (%) among all vessel segments analyzed. Brown color represents labeling of the endothelium with UEA-l and blue color COLL4-positive membranes. Type 1 vessels with an intact endothelium and basement membrane are the most common type in the study population, whereas type 2 vessels with irregularities restricted to the endothelium are rare. DWMLs express a high proportion of type 3 vessels with vascular bags. Likewise type 4 vessels with changes of both the endothelial cell layer and external collagenous membranes are common. (TIF 12345 kb) [file 40478_2018_632_MOESM5_ESM.tif]

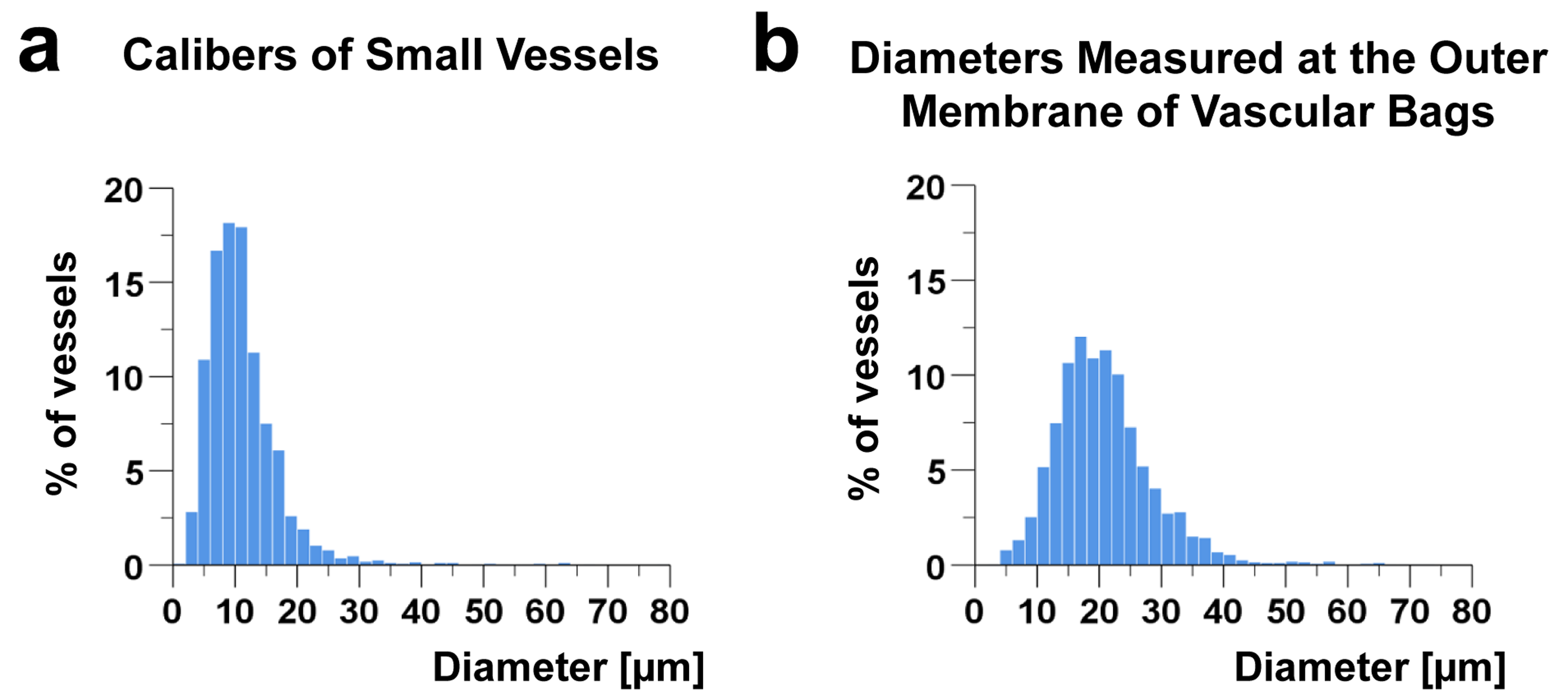

Supplement: Supplementary file 6 — Distribution of diameters. The diagram indicates the distribution of the diameters of small vessels (vessel calibers) (a) and maximum diameters at the outer membrane of the vascular bags (b). The diameters reported were determined in vessel segments that were in focus in images taken with the 20x objective and were used for quantitative analyses of vascular bags (see also Fig. 2). The width of the vascular bags was calculated as the difference between the two diameters (see also Fig. 3). (TIF 19068 kb) [file 40478_2018_632_MOESM6_ESM.tif]

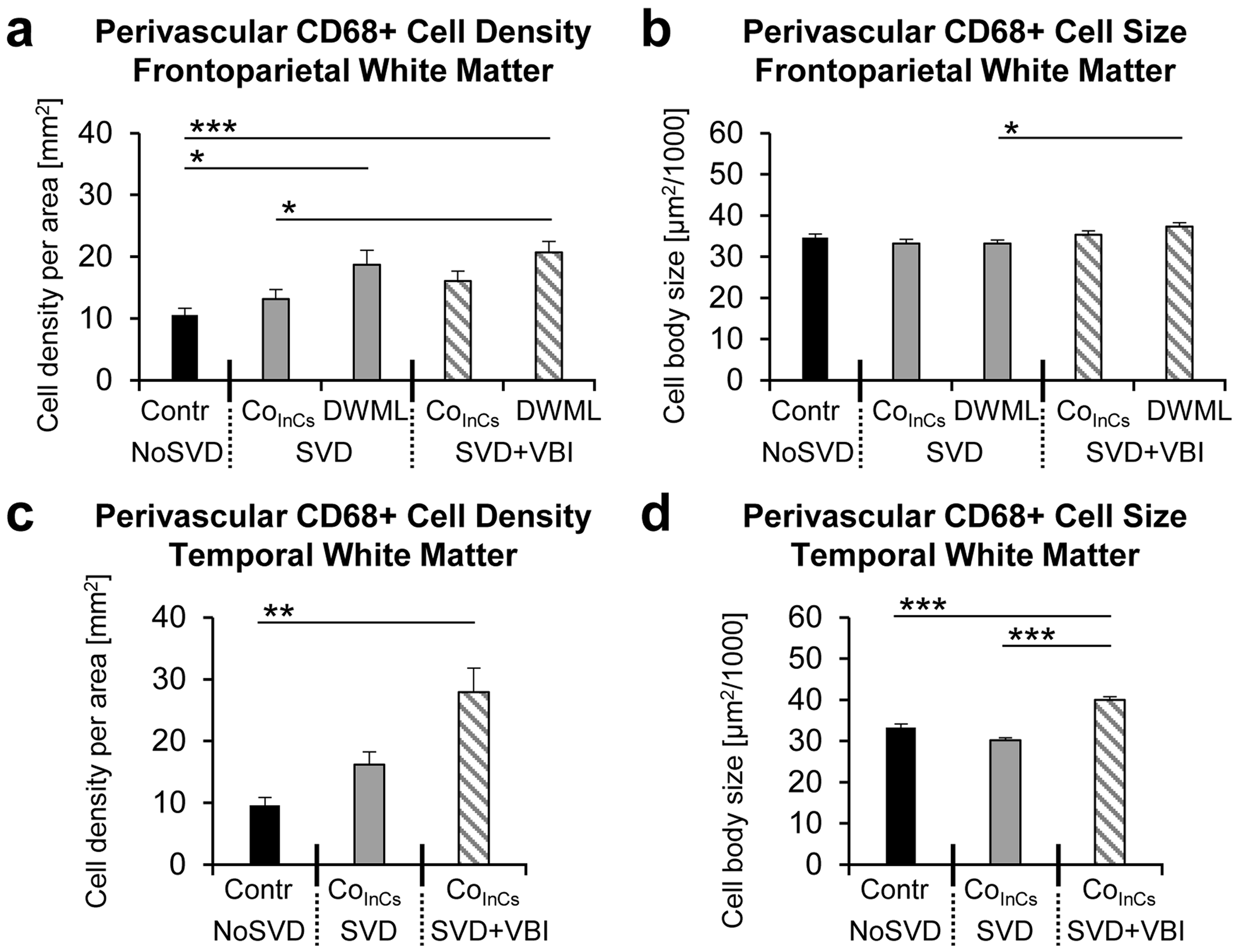

Supplement: Supplementary file 7 — Diagram of perivascular CD68-positive macrophages showing the density (a and c) and size (b and d) of cells in the frontoparietal (a-b) and temporal white matter (c-d) analyzed with three-way ANOVA (vascular disease x presence of DWML x white matter location) and the posthoc Games-Howell test. In the three-way ANOVA, the density of perivascular macrophages was significantly increased by vascular disease (F2,394 = 8.479, p = 0.004) and presence of DWMLs (F2,394 = 11.665, p = 0.001), and also depended on the white matter location (F1,394 = 9.135, p = 0.003). Moreover, the size of perivascular CD68-positive cells was significantly affected by vascular disease (F2,2856 = 65.003, p < 0.001) in interaction with the white matter location (F1,2856 = 19.668, p < 0.001), and the covariate age had a significant effect on the density (F1,394 = 65.231, p < 0.001) and size (F2,2856 = 9.527, p = 0.002) of the cells. a-b: In the frontoparietal white matter, posthoc analyses revealed a higher density of perivascular macrophages in DWMLs of all SVD cases (pure SVD, SVD + VBI) compared to NoSVD. Also, CD68-positive cells were significantly enlarged in the DWMLs of SVD + VBI cases compared to DWMLs in pure SVD. Neither the density nor the size of CD68-positive cells was significantly altered in the in-case control areas of SVD cases compared to the control white matter in NoSVD cases. c-d: In the temporal white matter, posthoc analyses indicated that the density of CD68-positive cells was significantly increased in-case control areas of SVD + VBI cases but not of pure SVD cases when compared to NoSVD controls. Notably, perivascular cells were significantly larger in DWMLs of SVD + VBI cases than in DWMLs of pure SVD cases or in the control white matter of NoSVD cases. The density or size of CD68-positive cells in in-case control areas in pure SVD was not altered when compared to NoSVD. * p < 0.05, ** p < 0.01, *** p < 0.001. (TIF 19710 kb) [file 40478_2018_632_MOESM7_ESM.tif]
